# Immunosenescence in aging and neurodegenerative diseases: evidence, key hallmarks, and therapeutic implications

**DOI:** 10.1186/s40035-025-00517-1

**Published:** 2025-11-27

**Authors:** Zhichun Chen, Zixu Mao, Weiting Tang, Yuxuan Shi, Jun Liu, Yong You

**Affiliations:** 1 Department of Neurology, The Second Affiliated Hospital of Hainan Medical University, Haikou, 570311 China; 2 Department of Neurology and Institute of Neurology, Ruijin Hospital Affiliated to Shanghai Jiao Tong University School of Medicine, Shanghai, 200025 China; 3 Departments of Pharmacology and Chemical Biology and Neurology, Emory University School of Medicine, Atlanta, GA 30322 USA; 4 Center for Neurodegenerative Diseases, Emory University School of Medicine, Atlanta, GA 30322 USA; 5 International Center for Aging and Cancer (ICAC), Hainan Medical University, Haikou, 571199 China; 6 Key Laboratory of Brain Science Research and Transformation in Tropical Environment of Hainan Province, Haikou, 571199 China

**Keywords:** Immunosenescence, Hallmarks, Brain aging, Neurodegenerative disease, Anti-aging therapy

## Abstract

Aging is a multifaceted biological process affecting various organ systems. Immunosenescence, a key feature of aging, markedly increases susceptibility to infections, cancers, autoimmune diseases, and also neurodegenerative disorders. Immunosenescence not only accelerates normal aging but also drives the progression of neurodegenerative diseases, including Alzheimer’s disease (AD) and Parkinson’s disease (PD). However, the lack of a consensus on the mechanistic hallmarks of immunosenescence presents a major barrier to the development and validation of anti-aging therapies. In this review, we propose 11 hallmarks of immunosenescence: genomic instability, telomere attrition, epigenetic dysregulation, stem cell exhaustion, loss of proteostasis, deregulated nutrient-sensing, mitochondrial dysfunction, cellular senescence, chronic inflammation, altered intercellular communication, and microbiome dysbiosis. We also elucidate the intricate interplay between immunosenescence and both normal brain aging and neurodegenerative pathologies, highlighting the pivotal involvement of age-related immune dysregulation in the pathogenesis of neurodegenerative disorders. This mechanistic connection is particularly evident in prototypical neurodegenerative conditions such as AD and PD, where immunosenescence appears to significantly contribute to disease progression and phenotypic manifestations. Given that the ultimate goal of immune aging research is to prevent or alleviate age-related diseases, we also discuss potential hallmark-targeting anti-immunosenescence strategies to delay or even reverse normal aging and neurodegeneration.

## Introduction

Aging is a primary risk factor for neurodegenerative diseases (NDDs), including Alzheimer’s disease (AD) and Parkinson’s disease (PD). Elucidating the mechanistic basis of aging and its downstream sequelae is essential for addressing age-related neurodegeneration. However, aging is a multidimensional process affecting multiple organ systems. The immune system, which is central to human physiology and intrinsically linked to neurodegenerative pathogenesis, provides us a strategic framework to elucidate how aging potentiates neurodegeneration. Immunosenescence drives systemic aging and represents a key therapeutic target for healthspan extension [1]. During aging, individuals face increased mortality risk from infections, strongly linked to immunosenescence [2]. Aging has systemic impacts on hematopoietic stem cells (HSCs), immune progenitors in the bone marrow (BM) and thymus, as well as mature immune cells across secondary lymphoid organs, blood, and tissues [3]. Immunosenescence is associated with accelerated organ aging [1], systemic inflammation [4], severe infection [5], impaired vaccine-induced immunity [6], and lower survival probability [7]. Critically, immunosenescence also alters the immunological responses to neurodegenerative pathologies in AD [8, 9], PD [10], and multiple sclerosis (MS) [11–13], thereby influencing progression of these diseases.

Despite broad clinical recognition, the mechanistic hallmarks of immunosenescence remain elusive. This review was aimed to: (1) characterize age-related immune remodeling and its core mechanistic hallmarks; (2) examine the contributions of immunosenescence to both brain aging and NDDs; and (3) propose hallmark-targeting strategies for immune rejuvenation and NDD intervention [14].

## Age-related changes in the immune system

The precise manifestations of immune aging across distinct anatomical compartments remain incompletely characterized. Critically, peripheral immunosenescence exhibits established yet mechanistically obscure links to NDDs, where dysregulation of myeloid trafficking, chronic inflammation, and impaired immunosurveillance accelerate neuropathology [15, 16]. A comprehensive understanding of both peripheral and central nervous system (CNS) immune alterations during aging can facilitate identification of both general and compartment-specific hallmarks of immunosenescence as well as their differential contributions to brain aging and neurodegeneration. In the following, we start by delineating age-related transformations across key immune compartments to better display the complex nature of immunosenescence.

### Immunosenescence in aged BM

**Fig. 1:**
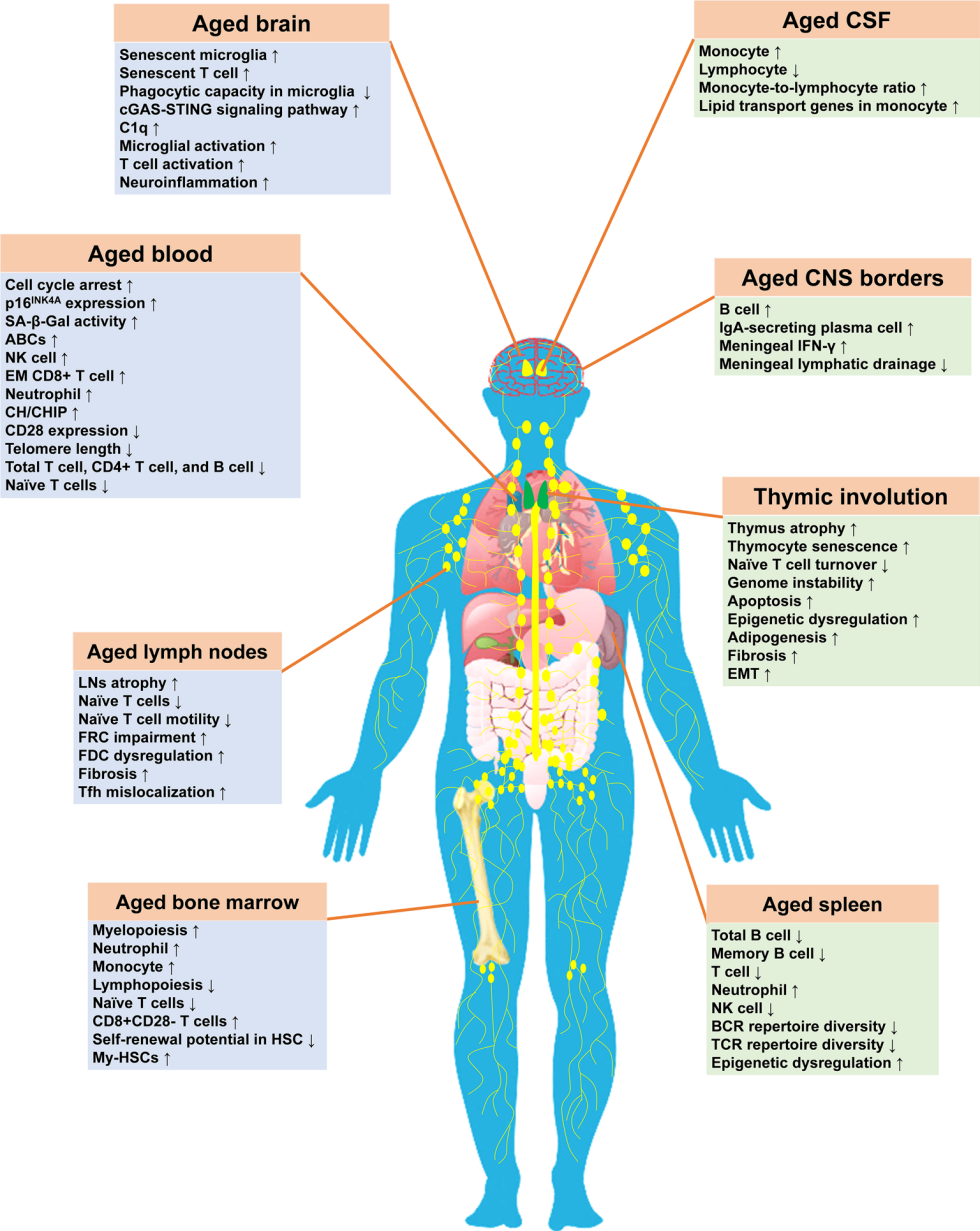
Age-related changes in the immune system. The impacts of immune aging are widespread, spanning from HSCs, immune progenitors, and naïve immune cells in the BM and thymus, to mature immune cells in secondary lymphoid nodes, spleen, blood, and other organs, such as brain. cGAS: cyclic GMP-AMP synthase; STING: stimulator of interferon genes; SA-β-Gal: senescence-associated β-galactosidase; CH: clonal hematopoiesis; CHIP: clonal hematopoiesis of indeterminate potential; EM: effector memory; ABCs: age-associated B cells; NK cell: natural killer cell; LNs: lymph nodes; FRC: fibroblastic reticular cell; FDC: follicular dendritic cell; Tfh cell: T follicular helper cells; My-HSCs: myeloid-biased hematopoietic stem cells; EMT: epithelial-to-mesenchymal transition; BCR: B cell receptor; TCR: T cell receptor

During aging, HSCs undergo substantial compositional and functional alterations, characterized by progressive losses of self-renewal capacity and regenerative potential [17]. Aging leads to a shift of HSC differentiation toward myelopoiesis (elevated neutrophils/monocytes) over lymphopoiesis, driven by increased myeloid-biased HSCs (my-HSCs) and reduced lymphoid-myeloid balanced HSCs (bal-HSCs) [18]. Concomitantly, the aged BM exhibits significant T cell pool remodeling, featured by naïve T cell depletion and accumulation of senescent CD8^+^CD28^−^ T cells [19]. Aging is also associated with reduced B cell lymphopoiesis in the BM [19]. Additionally, transcriptomic, metabolomic, and lipidomic profilings further revealed profound aging signatures in BM neutrophils [20], establishing neutrophil dysfunction as a key component of immunosenescence. Aging also affects the natural killer (NK) cell pool. NK cells demonstrate reduced proliferative capacity, immature phenotypes, and aberrant expression of stimulatory and inhibitory receptors during aging [21]. Collectively, these findings suggest extensive, coordinated alterations across the BM hematopoietic landscape during physiological aging (Fig. 1).

### Thymic involution

During fetal and neonatal development, the thymus rapidly grows, establishing the size and the diversity of the naïve T cell pool, while during puberty, thymus undergoes atrophy and declined activity [22]. Bohacova et al. (2024) demonstrated that the thymic output decreases with age, marked by a decline in CD38^+^ naïve T cells and an increase in CXCR3^hi^ naïve T cells in circulation [23]. The turnover of naïve T cells in the thymus exhibits an age-dependent decline as demonstrated by ^14^C measurements [24]. As a result, the reduced naïve T cell pool gradually leads to a deficiency in thymic function, despite the increased proliferation of activated naïve T cells [25]. This progressive degeneration fundamentally compromises adaptive immune renewal, positioning thymic involution as a defining feature of immunosenescence (Fig. 1).

### Immunosenescence in aged spleen

During aging, the spleen also undergoes extensive changes (Fig. 1). Spleens of aged rats exhibit reductions of B cells, T cells, granulocytes, and macrophages across lymphoid follicles, marginal zones, and periarterial sheaths compared to young rats [26]. Aging also leads to decreased B cell receptor (BCR) H-CDR3 repertoire diversity in splenic B/memory B cells [27], and a decline of CD4^+^ T cell receptor (TCR) diversity due to sporadic clonal expansions with advancing age [28]. These coordinated cellular and receptor landscape alterations critically compromise adaptive immune functionality, positioning the spleen as a pivotal contributor to age-related immune decline.

### Immunosenescence in aged lymph nodes (LNs)

Aged LNs exhibit impaired cellular migration, disruption of immune cell localization, dysregulation of cytokine/chemokine production, and diminished adaptive responses due to the breakdown of naïve T/B cell homeostasis [29]. Notably, aged LNs show reductions of CD4^+^ T cells, plasmacytoid dendritic cells, and NK cells (Fig. 1) [30]. The timing of atrophy also varies: the skin-draining LNs shrink by 6–9 months in mice, while the deeper tissue-draining LNs undergo atrophy by 18–20 months [31]. Stromal deterioration is a critical mechanism underlying LN atrophy. Fibroblastic reticular cells form the reticular network within the LNs, along which the LN naïve T cells from the blood stream crawl to search for dendritic cells. Aging of the fibroblastic reticular cells impairs naïve T cell motility and survival signals [32], while aging-associated impairment of follicular dendritic cells contributes to poor antigen retention and decreased production of high-affinity antibodies [33]. These structural and functional declines in stromal cells and immune cells within the LNs collectively impair pathogen clearance and vaccine efficacy through erosion of critical immune surveillance mechanisms.

### Immunosenescence in aged blood

The percentages of total T cells, CD4^+^ T cells, and B cells are reduced in the peripheral blood of older individuals [34, 35], whereas the NK cell and late differentiated T cell populations are increased [34, 35]. The T cell pool undergoes extensive remodeling during aging [36]. The most prominent changes are the decline in the naïve T cell pool and the expansion of memory-like T cells [36]. Recently, Wang et al. (2025) found dramatical declines of naïve T cell counts and TCR repertoire diversity with advancing age in humans, further providing new insights into the aging of T cell populations [37]. Concurrently, the accumulation of age-associated B cells (ABCs) is another crucial immune feature during aging. ABCs are considered a type of memory B cells that express the transcription factor T-bet and the integrins CD11b and CD11c [38, 39]. ABCs significantly inhibit the growth and/or survival of pro-B cells and common lymphoid progenitors, thereby impairing B lymphopoiesis and accelerating B cell aging [40, 41]. During normal aging, the neutrophil counts in peripheral blood are mildly reduced [42]. In addition, they also display age-dependent alterations, such as impaired migration and dysregulated pro-inflammatory responses [43]. Systemically, the blood immune cells in the elderly population present several senescent markers, such as CD28 loss [34], elevated senescence-associated β-galactosidase (SA-β-Gal) activity [44], increased telomere attrition and cell cycle arrest (Fig. 1) [44]. These aging-associated changes are mostly observed in CD8^+^ T effector memory (TEM) cells, which exhibit senescent features possibly due to telomere shortening and enhanced p16^INK4A^ expression [44]. The TEM cells also show a specific gene expression signature previously observed in senescent fibroblasts and exhibit a unique senescence-associated secretory phenotype (SASP) comprising diverse cytokines, chemokines, and extracellular matrix proteases [45].

### Immunosenescence at the CNS borders

The meninges host immune cells that provide immunosurveillance of the CNS, such as IgA-secreting plasma cells [46] and T cells [47]. B cells in the meninges originate from the local calvaria, which provides a BM niche for hematopoiesis [48]. Particularly, in aged mice, antigen-experienced B cells can migrate from peripheral blood into the meninges [48]. Moreover, the IgA-secreting plasma cells are situated near the dural venous sinuses and their numbers significantly increase with age [46]. T cells within the dural sinuses can recognize cerebrospinal fluid (CSF)-derived antigens, initiating adaptive immune responses in the dural meninges [47]; however, the impact of aging on these T cells remains poorly understood. Additionally, aging is associated with a decline in meningeal lymphatic drainage, leading to the accumulation of toxic, misfolded proteins in the CNS [49, 50]. Border-associated macrophages (BAMs) are crucial immune subpopulations in the meningeal compartments of the brain, such as the dura mater and subdural spaces. These macrophages are specialized immune cells that exhibit unique transcriptional profiles tailored to their specific locations and functions [51]. Future studies are required to understand how aging compromises the functional integrity of BAMs.

### Immunosenescence in brain parenchyma and CSF

Microglial function declines with aging, characterized primarily by increased oxidative stress due to mitochondrial dysfunction [52] and impaired phagocytosis [53]. Olah et al. (2018) identified a series of genes preferentially expressed by aged microglia in the human brain, termed the HuMi_Aged gene set, confirming the existence of aged microglia phenotype in human brain [54]. Subsequent studies revealed extensive microglial transcriptomic remodeling [55] and divergent glial activation patterns during aging [56]. Notably, Rachmian et al. (2024) identified a subpopulation of senescent microglia expressing high levels of triggering receptor expressed on myeloid cells 2 (TREM2), which is linked to impaired cognition and increased neuroinflammation during normal aging [9]. Moreover, microglia in the white matter cluster in nodules during white matter aging, and are engaged in the clearance and repair of degenerated myelin [57]. Beyond microglia, other immune cells within the brain parenchyma also exhibit significant changes with age. It has been shown that T cells are clonally expanded in aged brains, representing a senescent phenotype distinct from that in aged blood, suggesting that they may experience specific CNS antigens [58]. Astrocytes also play a crucial role in CNS immunity and neuroinflammation [59]. Lee et al. (2022) identified an aged astrocyte subtype in the hippocampus, which exhibits abnormal accumulation of autophagosomes, impairing protein trafficking and secretion [60], highlighting the role of astrocytes in CNS aging and disease pathology. Immunosenescence in the brain parenchyma is also involved in the alterations of age-related immune signaling pathways. During brain ageing, the cyclic GMP-AMP synthase (cGAS)-stimulator of interferon genes (STING) signaling pathway is activated due to DNA damage, driving chronic neuroinflammation in the CNS [61]. Additionally, receptor-interacting protein kinase 1 (RIPK1) is activated during brain aging through suppression of transforming growth factor-β-activated kinase 1 signaling, leading to excessive neuroinflammatory responses [62].

There are limited studies on the immune phenotypes related to aging in the CSF. A recent study found that aging is associated with higher monocyte counts, lower lymphocyte counts, and a higher monocyte-to-lymphocyte ratio [63], along with upregulated expression of lipid transport genes in monocytes in aged CSF [64]. Despite these insights, the full spectrum of immunosenescence in the CSF remains poorly understood, necessitating extensive research in the future.

## Hallmarks of immunosenescence

**Fig. 2:**
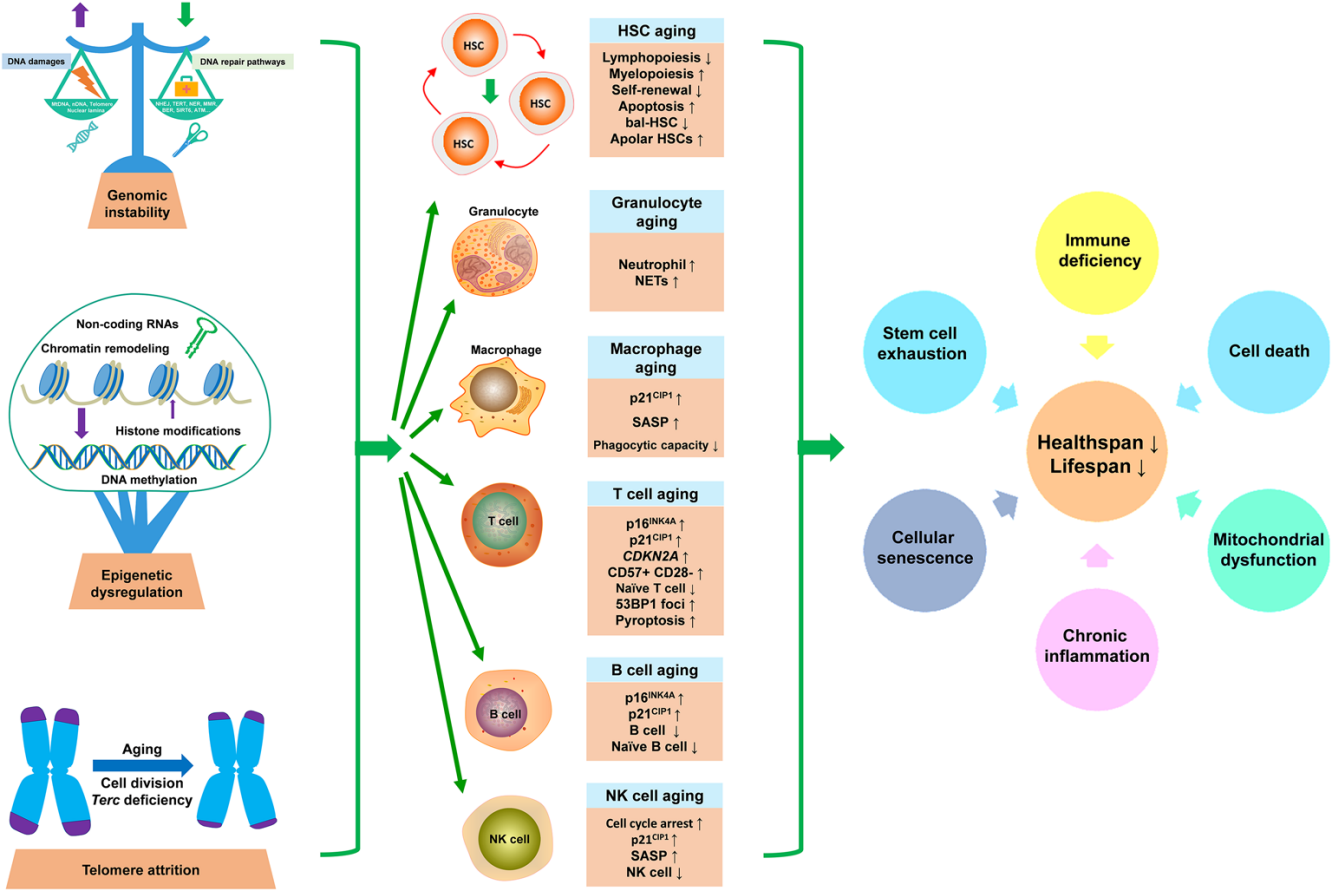
Genomic instability, telomere shortening, and epigenetic dysregulation can dramatically reshape the gene expression patterns of immune cells, thereby inducing immune cell aging and leading to subsequent cellular malfunction and helathspan/lifespan decline. HSCs: hematopoietic stem cells; mtDNA: mitochondria DNA; nDNA: nuclear DNA; NHEJ: non-homologous DNA end joining; TERT: telomerase reverse transcriptase; NER: nucleotide excision repair; MMR: mismatch repair; BER: base excision repair; ATM: ataxia telangiectasia mutated; NET: neutrophil extracellular trap; SASP: senescence-associated secretory phenotype; CDKN2A: Cyclin Dependent Kinase Inhibitor 2A; 53BP1: p53-binding protein 1; NK cell: natural killer cell

**Fig. 3:**
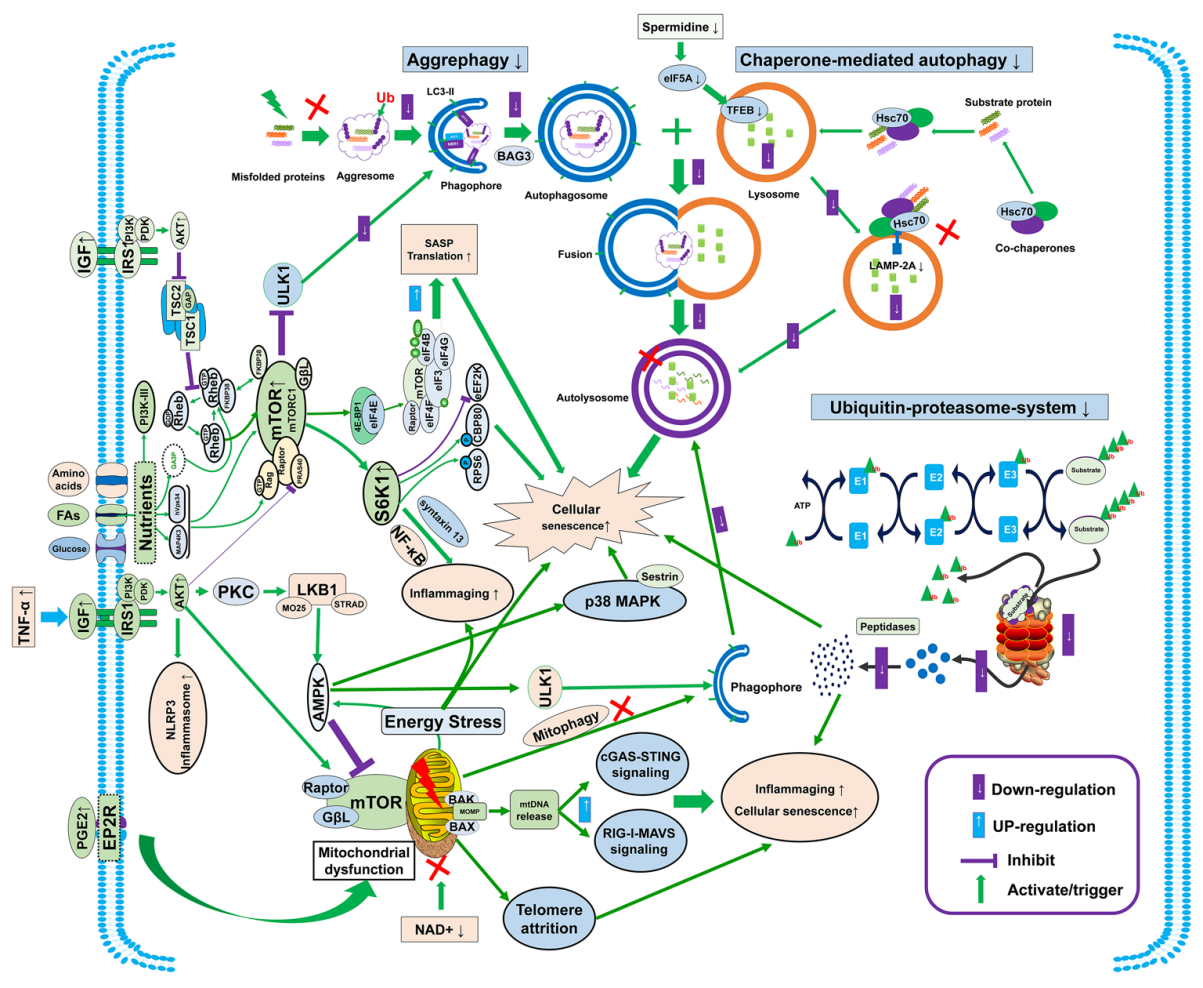
Loss of proteostasis, deregulated nutrient-sensing, and mitochondrial dysfunction synergistically cause bioenergetic dysmetabolism and quality control impairment, thereby inducing multiple immune aging phenotypes such as inflammaging, cellular senescence, SASP, telomere attrition, and NLRP3 inflammasome activation. SASP: senescence-associated secretory phenotype; Ub: ubiquitin; LC3-II: microtubule-associated protein 1 light chain 3-II; eIF5A: eukaryotic initiation factor 5A; TFEB: transcription factor EB; BAG3: Bcl2-associated athanogene 3; LAMP-2A: lysosomal associated membrane protein-2A; mTOR: mechanistic target of rapamycin; TSC: tuberous sclerosis complex; IGF: insulin-like growth factor; IRS1: insulin receptor substrate 1; PI3K: phosphoinositide 3-kinase; PDK: phosphoinositide-dependent kinase 1; AKT1: protein kinase B; ULK1: UNC-51-like kinase 1; FAs: fatty acids; 4E-BP1: eukaryotic translation initiation factor 4E-binding protein 1; FKBP38: FK506-binding protein38; mTORC1: mechanistic target of rapamycin complex 1; GβL: G protein β-like; eIF4E: eukaryotic translation initiation factor 4E; S6K1: ribosomal protein S6 kinase beta 1; NF-kB: nuclear factor-κB; PKC: protein kinase C; LKB1: liver kinase B1; AMPK: AMP-activated protein kinase; NLRP3: NOD-, LRR- and pyrin domain-3; BAK: BCL2 antagonist/killer; BAX: Bcl-2 associated X-protein; NAD^+^ : nicotinamide adenine dinucleotide; p38 MAPK: mitogen-activated protein kinase; cGAS-STING: cyclic GMP-AMP synthase-stimulator of interferon genes; RIG-I-MAVS: retinoic acid-inducible gene I-mitochondrial antiviral signaling protein; RPS6: ribosomal protein S6; CBP80: Cap binding protein 80; eEF2K: eukaryotic elongation factor 2 kinase

**Fig. 4:**
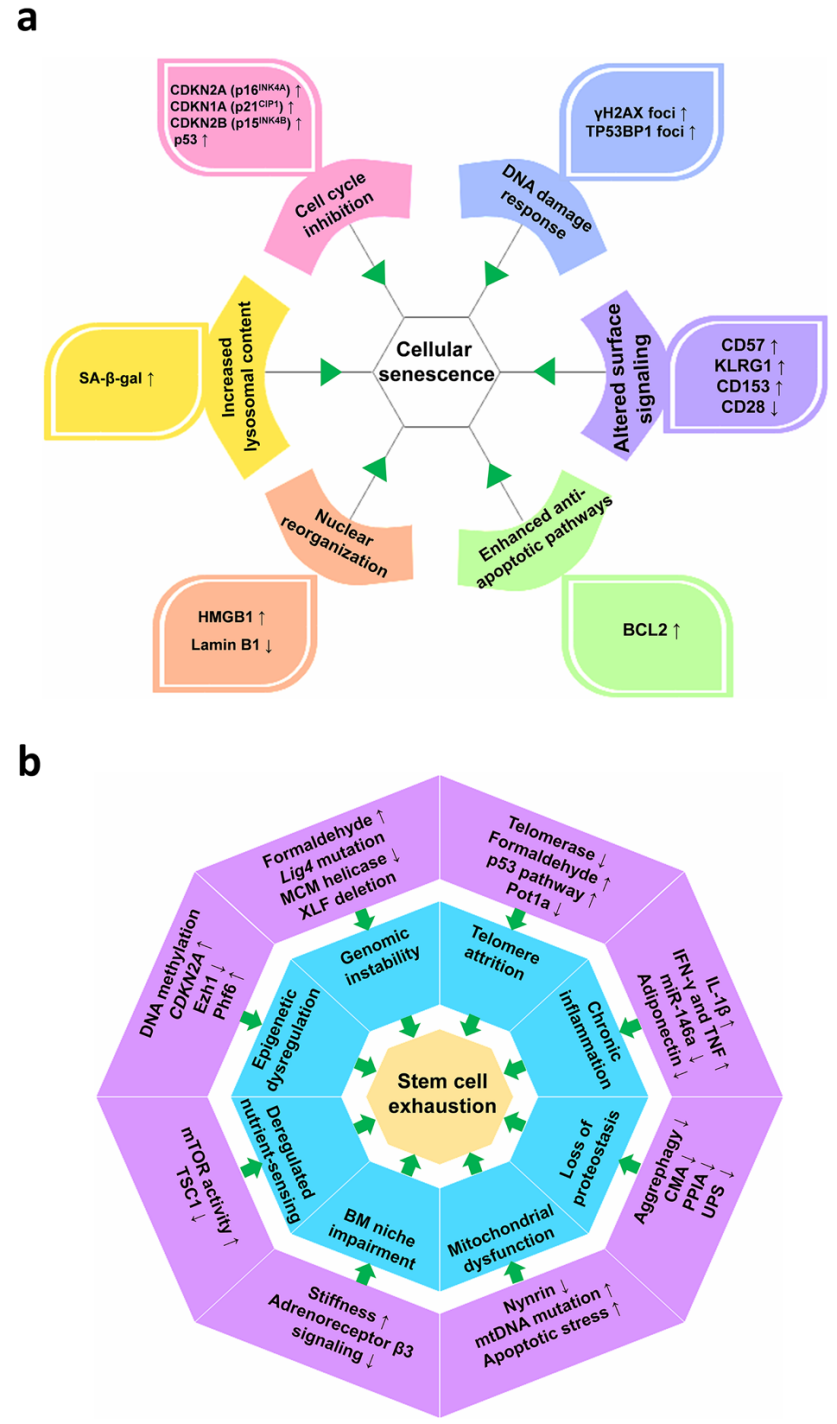
Hallmarks of cellular senescence and stem cell exhaustion. **a** Senescence hallmarks in immune cells may include cell cycle inhibition, DNA damage response, altered cell surface signaling, increased lysosomal content, nuclear reorganization, and enhanced anti-apoptotic pathways. SASP is not included here due to its profound overlap with inflammatory and immune responses. **b** Multiple molecular hallmarks, such as genomic instability, telomere attrition, epigenetic dysregulation, chronic inflammation, mitochondrial dysfunction, and loss of proteostasis, contribute to HSC aging. SASP: senescence-associated secretory phenotype; CMA: chaperone-mediated autophagy; PPIA: peptidyl-prolyl isomerase A; UPS: ubiquitin–proteasome-system; mTOR: mechanistic target of rapamycin; TSC1: tuberous sclerosis complex 1; CDKN2A/2B/1A: cyclin dependent kinase inhibitor 2A/2B/1A; Phf6: plant homeodomain 6; MCM: mini-chromosome maintenance; XLF: XRCC4-like factor; BM: bone marrow; SA-β-Gal: senescence-associated β-galactosidase; HMGB1: high mobility group box 1; BCL2: B-cell lymphoma-2; TP53BP1: tumor protein P53-binding protein 1; KLRG1: killer cell lectin-like receptor G1

**Fig. 5:**
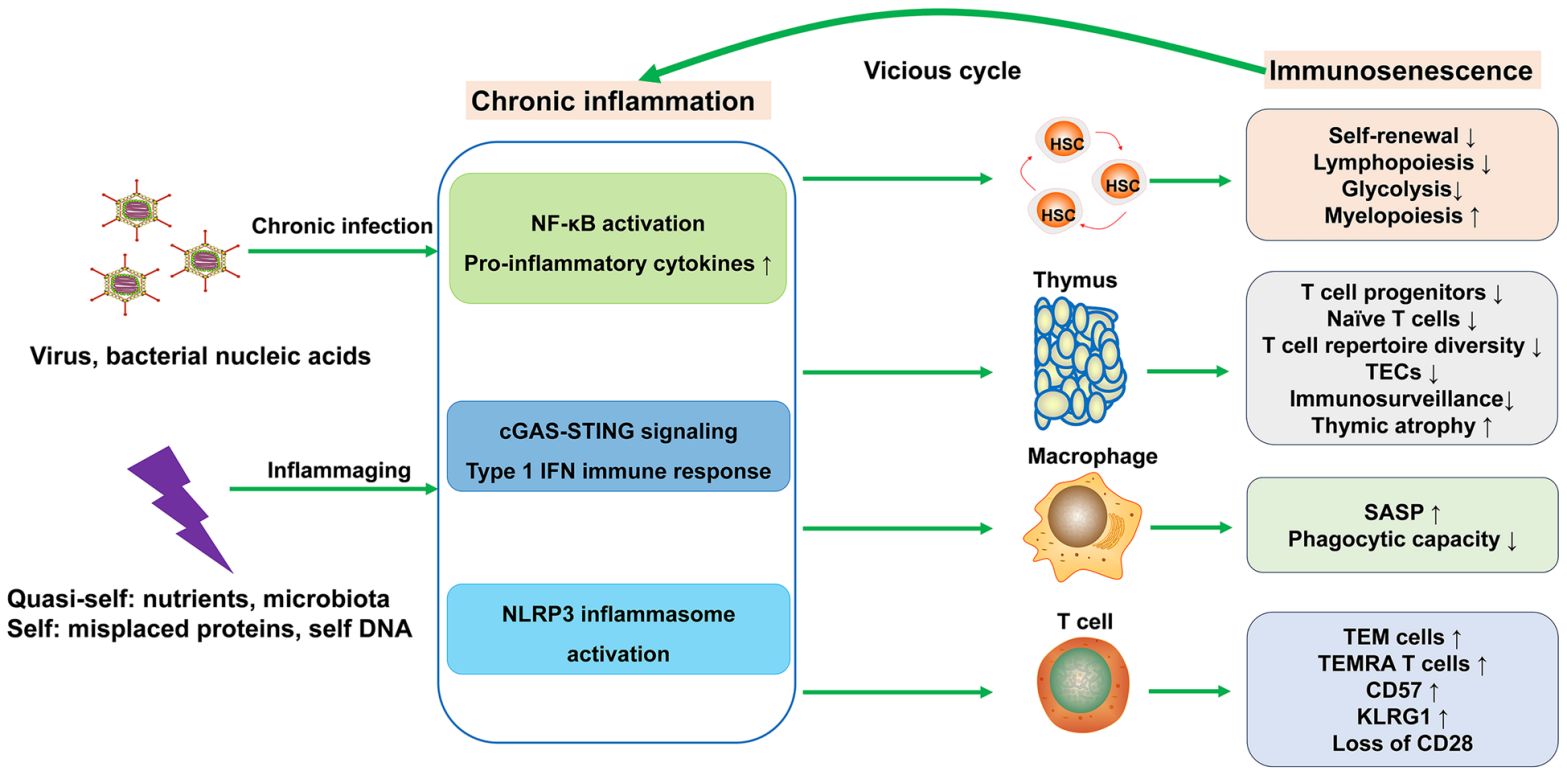
Chronic inflammation as a key component of the aged immune system. In the progression of immune aging, chronic inflammation manifests through two distinct mechanisms: non-sterile, pathogen-driven immune responses and sterile, low-grade inflammation, commonly referred to as ‘inflammaging’. Both mechanisms converge to activate several pro-inflammatory molecular pathways, such as the cGAS-STING signaling pathway, which is increasingly recognized as a central mediator of age-related immune dysfunction. SASP: senescence-associated secretory phenotype; KLRG1: killer cell lectin-like receptor G1; HSC: hematopoietic stem cell; NF-kB: nuclear Factor kappa B; cGAS-STING: cyclic GMP-AMP synthase-stimulator of interferon genes; NLRP3: NOD-, LRR-, and pyrin domain-containing protein 3; TEC: thymic epithelial cells

**Fig. 6:**
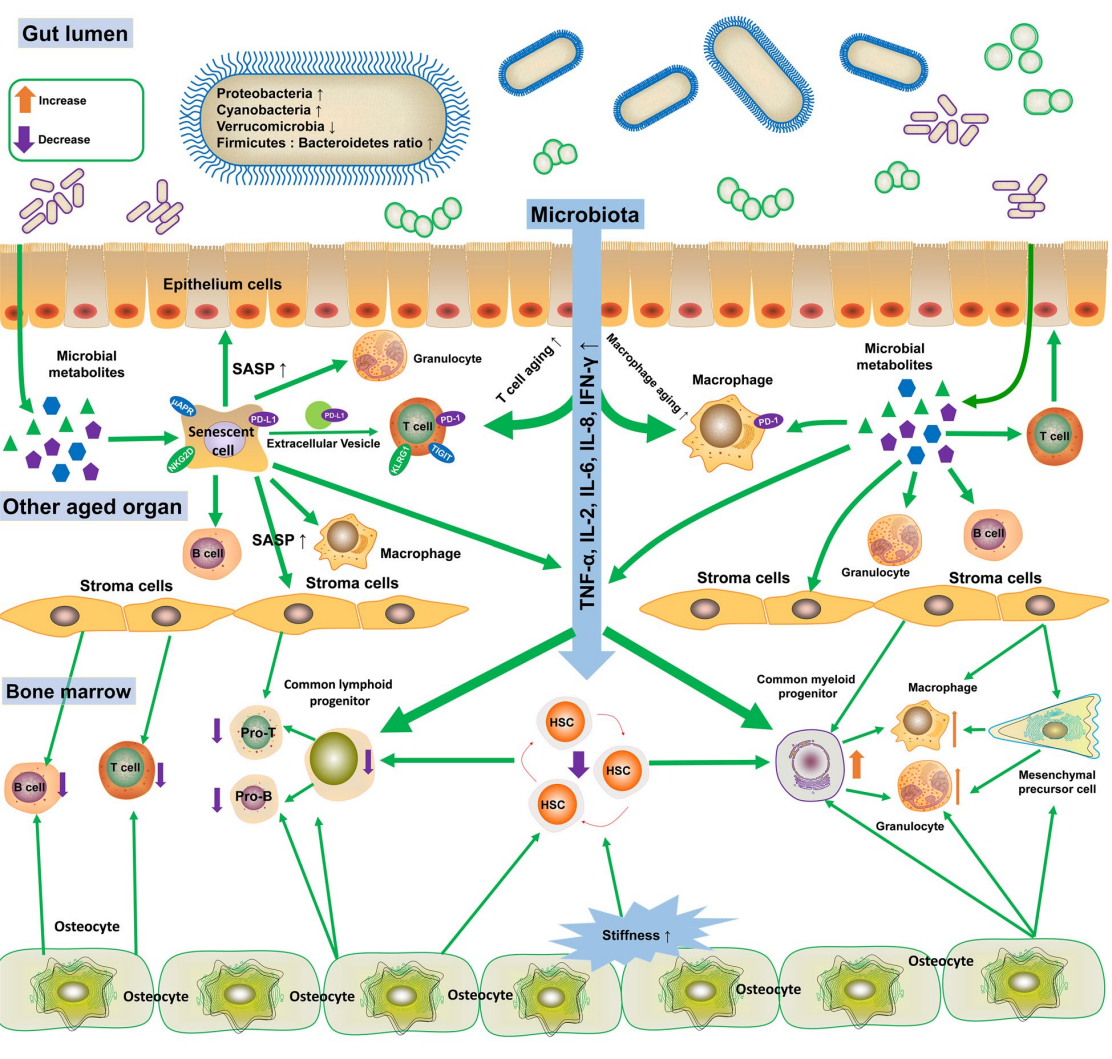
Aberrant intercellular communication and microbiome dysbiosis jointly disrupt the intercellular signaling networks within the whole body, thereby amplifying pro-inflammatory responses and leading to multiple immune aging phenotypes, including impaired self-renewal capacity of HSCs, increased myelopoiesis, reduced lymphopoiesis, SASP, and immune cell senescence. SASP: senescence-associated secretory phenotype; PD-1: programmed death-1; PD-L1: programmed death ligand 1; TIGIT: T-cell immunoreceptor with immunoglobulin and immunoreceptor tyrosine-based inhibitory motif domain; KLRG1: killer cell lectin-like receptor G1; NKG2D: natural killer (NK) group 2 member D; uAPR: urokinase-type plasminogen activator receptor; HSC: hematopoietic stem cell

In 2013, López-Otín et al. proposed nine hallmarks of aging [65]. In 2023 and 2025, three and two new hallmarks were introduced respectively, providing new insights into the fundamental mechanisms of the aging process [66, 67]. Currently, the molecular drivers of immunosenescence remain incompletely characterized. Here, we propose 11 mechanistic hallmarks of immunosenescence to better elucidate its underlying mechanisms, including genomic instability (Fig. 2), telomere attrition (Fig. 2), epigenetic dysregulation (Fig. 2), loss of proteostasis (Fig. 3), deregulated nutrient-sensing (Fig. 3), mitochondrial dysfunction (Fig. 3), stem cell exhaustion (Fig. 4), cellular senescence (Fig. 4), chronic inflammation (Fig. 5), altered intercellular communication (Fig. 6), and microbiome dysbiosis (Fig. 6).

### Genomic instability

DNA damage, particularly point mutations, single-strand breaks and double-strand breaks (DSBs), are particularly prevalent among aged immune cells [68], positioning genomic instability as a fundamental driver of immunosenescence (Fig. 2) [69]. Studies in laminopathies such as Hutchinson-Gilford progeria syndrome (which is caused by *LMNA* truncations) have demonstrated that lamina defects can induce premature aging phenotypes [70]. Notably, *Lmna*-mutant mice exhibit accelerated immunosenescence [71]. Similarly, Lamin B1 reduction specifically drives immunosenescence in *Drosophila* fat bodies [4]. Multiple molecular mechanisms are involved in the repair of DSBs during DNA damage in order to maintain genome integrity and cellular homeostasis, including non-homologous end joining [65, 66]. Age-dependent impairment of non-homologous end joining-mediated DNA break repair in aged B cells [72] and deficient activity of DSB repair nuclease MRE11A in T cells [73] directly implicate DNA repair dysfunction in immunosenescence. The deficiency of Ercc1, a DNA excision repair protein, in humans or mice has been shown to precipitate premature aging through genotoxic stress [74]. Peripheral lymphocytes isolated from 4- to 5-month-old *Ercc1*^−/∆7^ mice exhibit 15-fold enhanced expressions of senescent markers p16^INK4A^ and p21^CIP1^, comparable to the levels in 2.5-year-old wild-type mice, confirming premature immunosenescence [74]. In addition, *Ercc1* deficiency further drives the accumulations of aged Tregs [75] and microglia [76], underscoring its systemic impact. Similarly, suppression of the DSB nuclease MRE11A induces T cell senescence, while its overexpression reverses T cell senescence phenotypes [73]. The MRE11 complex component Nbs1, encoded by *NBS1*, proves equally vital: young patients with *NBS1* mutations display senescent CD57^+^CD28^−^ T cells [77] and macrophage-specific *NBS1* deficiency is sufficient to trigger cellular senescence and aberrant inflammation [78]. Consistently, ataxia telangiectasia mutated (ATM) deficiency impairs DSB repair, promoting CD4^+^ T cell senescence and apoptosis [79]. Collectively, these findings establish DSB repair failure as a core mechanism of immunosenescence (Fig. 2).

### Telomere attrition

Telomere attrition constitutes a defining hallmark of immune aging (Fig. 2). Wild-type mice with short telomeres exhibit hematopoietic and immune defects resembling immunosenescence [80], characterized by B and T cell lymphopenia in the spleen [80]. Moreover, telomerase-deficient (*Terc*^−/−^) mice exhibit disrupted hematopoietic niches that impair B lymphopoiesis and promote myeloproliferation [81], alongside systemic CD4^+^ T cell reduction and naïve T cell depletion across the thymus, blood, and spleen [82]. Furthermore, disruption of telomere integrity by KML001 induces T cell senescence [83, 84]. Crucially, Mendelian randomization analysis has confirmed causal links between telomere shortening and functional decline in human T and B cells [85], collectively establishing telomere dynamics as a fundamental regulator of immunosenescence.

### Epigenetic dysregulation

Epigenetic dysregulation is causally linked to the aging process [86], yet its specific impacts on immunosenescence remain poorly characterized. During aging, HSCs undergo extensive remodeling of epigenetic landscape to maintain lifelong production of peripheral blood cells, especially immune cells (Fig. 2). This involves DNA methylation changes at key regulatory sites: increased methylation at differentiation-promoting transcription factor-binding sites versus decreased methylation of HSC maintenance genes [87]. Hidalgo et al. (2012) showed that deletion of the polycomb group protein Ezh1 triggers HSC depletion by modifying monomethylation and dimethylation of H3K27 [88]. In addition to Ezh1, the chromatin regulator plant homeodomain 6 (Phf6) also plays a key role in the regulation of HSC aging. Deletion of Phf6 attenuates senescent hallmarks in aged murine HSCs through epigenetic mechanisms [89]. The nicotinamide adenine dinucleotide (NAD^+^)-dependent deacetylase SIRT1 is down-regulated broadly during aging across immune compartments, spanning from hematopoietic stem and progenitor cells to CD8^+^CD28^−^ T cells [90]. Mechanistically, SIRT1 deficiency induces IRF3/IRF7 hyperacetylation in the DNA-binding domain, which inhibits liquid–liquid phase separation and antiviral immunity [91], establishing histone acetylation dysregulation as a key immunosenescence mechanism. During aging, microRNA expression shows substantial alterations across naïve, central memory, and CD8^+^ TEM cell subpopulations, implicating a role of microRNAs in the regulation of immunosenescence [92]. Elevated miR-21 in older individuals leads to the activation of senescence-promoting transcription factor networks, such as mitogen-activated protein kinase (MAPK) signaling pathways [93], while reduced miR-181a impairs the T cell-mediated antiviral immunity—recapitulating human immune aging in murine models [94]. These findings underscore miR-21 and miR-181a as critical regulators of T cell senescence and broader immunosenescence.

### Loss of proteostasis

Proteostasis refers to the maintenance of a healthy and balanced proteome within cells, and involves various molecular processes, especially protein degradation. Unlike most cells dependent on the ubiquitin–proteasome system (UPS), the HSCs preferentially utilize aggrephagy to clear misfolded protein monomers [95]. Importantly, aggrephagy dysfunction due to Bag3 deficiency has been shown to trigger protein aggregation, impair self-renewal capacity, and promote myeloid-biased differentiation of HSCs [95]. Additionally, chaperone-mediated autophagy (CMA), which is responsible for lysosomal protein degradation [96], declines with age in murine HSCs (Fig. 3), contributing to HSC pool depletion [97]. Autophagy is essential for maintaining T cell homeostasis, activation, and differentiation [98]. Age-dependent autophagy dysfunction shapes T cell aging. Age-related decrease of spermidine, an endogenous autophagy-inducing metabolite, drives autophagy defects (Fig. 3) through dysregulation of eukaryotic initiation factor 5A (eIF5A) and transcription factor EB (TFEB), thereby inducing T cell aging [99]. Moreover, CMA activity decreases in aged T cells due to the failure of efficient upregulation of lysosome-associated membrane protein type 2A (LAMP-2A) expression in response to TCR engagement, leading to impaired immune function of T cells. This indicates that CMA also regulates T cell aging (Fig. 3) [100]. In addition, UPS impairment contributes to T cell senescence. Suppression of proteasome induction in CD4^+^ T cells upon TCR stimulation leads to T cell senescence [101]. Specifically, proteasome dysfunction in CD4^+^ T cells induces senescence-associated phenotypes, such as impaired proliferation, age-related cytokine production and elevations of CD4^+^PD-1^+^CD44^High^ T cells [101]. Similarly, B cells experience autophagic capacity decline with age, which compromises the functions of memory B cells [102]. This deterioration stems partly from age-related reduction of spermidine and insufficient TFEB expression (Fig. 3) [102]. This confirms that impaired autophagy caused by deficient TFEB activation is a key mechanism of B cell senescence [102]. Autophagy dysfunction also promotes senescence-associated microglia, which are characterized by reduced proliferation, increased CDKN1A/p21^CIP1^, dystrophic morphologies and SASP [103]. Moreover, autophagy dysfunction causes aging-related phenotypes in macrophages [104, 105], further highlighting the role of autophagy in regulating immune aging.

### Deregulated nutrient-sensing

During nutrient-sensing processes, extracellular ligands, such as insulin/insulin growth factors (IGFs), activate intracellular cascades including the phosphoinositide 3-kinase (PI3K)–protein kinase B (PKB, also known as serine/threonine kinase AKT)–mTOR pathway through interacting with receptor tyrosine kinases on cell surface [66]. mTOR functions as a master sensor responding to nutrients such as glucose and amino acids, and to stressors such as hypoxia, and energy stress [106]. It coordinates translation, lipid synthesis, lysosomal biogenesis, and growth signaling by phosphorylating key effectors (AKT, S6K, 4EBP1, TFEB) in these pathways. The growth hormone (GH)–insulin-like growth factor-1 (IGF1) axis regulates both development and aging processes by activating the PI3K–AKT–mTOR complex-1 (mTORC1) signaling. GH receptor activation promotes NOD-, LRR- and pyrin domain-containing protein 3 (NLRP3) inflammasome formation in macrophages, accelerating immune aging; conversely, GH receptor deficiency preserves naïve T cells and suppresses inflammaging [107].

Similarly, the insulin–IGF1 signaling suppresses anti-aging transcription factors DAF-16/FOXO and heat-shock transcription factor 1 (HSF1) in *C. elegans* [108] and contributes to immune aging in mammals through the JAK/AKT-mediated STAT3 activation [109]. The p110δ subunit of PI3K is encoded by *PIK3CD*, which is specifically expressed in leukocytes and is required for the maintenance of lymphocyte homeostasis. Gain-of-function mutations of *PIK3CD* cause naïve T cell deficiency and accumulation of senescent effector T cells in humans—phenotypes that can be partially rescued by mTOR inhibition [110]. Notably, both CD8^+^ T cells and NK cells in patients with *PIK3CD* gain-of-function mutations show reduced immune capacity to kill EBV-infected B cells [111], confirming a central role of PI3K in the regulation of immunosenescence.

mTOR plays a key role in the occurrence of immunosenescence. mTOR controls SASP by regulating the translation of MK2 kinase through 4EBP1 (Fig. 3). mTOR inhibition prevents the induction of SASP [112]. Activation of mTOR in the HSCs of young mice causes increased mRNA expression of p16^INK4A^, p19^ARF^, and p21^CIP1^, decreased lymphopoiesis, and impairment of self-renewal, similar as phenotype of HSCs from aged mice [113]. Consistently, mice expressing active mutant variants of RagC, a key downstream mediator of mTORC1 complex, display multiple features of immune aging, such as cellular senescence, SASP, increased inflammaging, and a ~ 30% reduction in lifespan [114]. Conversely, mTOR inhibition by rapamycin extends the lifespan of aged mice and restores HSC self-renewal capacity and function [113]. Additionally, genetic deletion of S6K1, a core mTOR signaling component, extends lifespan and mitigates age-related pathologies including immune dysfunction [115], suggesting the mTOR–S6K1 pathway as a core regulator of immunosenescence (Fig. 3). In contrast, the mTORC1–TFEB axis attenuates aging phenotypes by suppressing DNA damage accumulation, enhancing autophagic clearance, and inhibiting cellular senescence [116]. Age-related spermidine depletion decreases TFEB expression and compromises TFEB-mediated autophagy in B cells [102], while spermidine supplementation induces TFEB expression and restores B cell response in old mice. This finding establishes that the mTORC1–TFEB signaling is a key target for mitigating immunosenescence through lysosomal-autophagic regulation [102].

AMP-activated protein kinase (AMPK) is a key metabolic regulator that maintains cellular energy balance. AKT inhibits AMPK activity through direct phosphorylation and indirect pathways, promoting anabolic processes while suppressing catabolic responses. AMPK activation extends lifespan, possibly by inducing autophagy through mTOR and UNC-51-like kinase 1 signaling [117]. While AMPK signaling plays an essential role in T cell metabolism [117], whether it also regulates T cell aging remained undefined until recent mechanistic insights. Callender et al. (2018) showed that AMPK can regulate p38 MAPK signaling, which induces SASP in senescent CD8^+^ T cells [45]. Crucially, AMPK-TAB1 activates p38 to induce human T cell senescence, characterized by reduced telomerase activity and T cell proliferation (Fig. 3) [118], while blockade of AMPK-TAB1-dependent p38 activation reverses the proliferative defects of senescent T cells [118]. Together, AMPK may work in concert with p38 MAPK to induce T cell senescence.

### Mitochondrial dysfunction

Normal mitochondrial activity is required for the survival of HSCs, myeloid progenitors, and B-lymphoid progenitors [119]. Mitochondrial impairment compromises the dormancy, regenerative capacity, lineage commitment, and self-renewal potential of HSCs [120–122]. Accumulation of mitochondrial DNA (mtDNA) damage, frequently resulting from defective mtDNA proofreading by DNA polymerase γ, is a key molecular driver of premature hematopoietic aging [122]. Following mtDNA DSB formation, BAK and BAX proteins promote mitochondrial outer membrane permeabilization during cellular senescence, allowing mtDNA release into the cytosol [123, 124]. This induces a retinoic acid-inducible gene I (RIG-I)–mitochondrial antiviral signaling (MAVS)-dependent immune response [125] and activates the cGAS-STING signaling pathway (Fig. 3) [123]. In aged primary T cells, mitochondrial dysfunction induces telomere attrition, a canonical senescence driver (Fig. 3) [126]. Moreover, targeted deletion of mitochondrial transcription factor A (*TFAM*) exacerbates T cell senescence, precipitating metabolic dysregulation, cognitive decline, and premature mortality [127]. The lipid messenger prostaglandin E2 (PGE_2_) released by senescent cells is an essential inducer of mitochondrial dysfunction during aging [128]. PGE_2_ promotes the acquisition of senescence markers in activated CD8^+^ T cells, such as increased p16 expression, loss of CD28 expression and telomere shortening (Fig. 3) [129]. NAD^+^, an essential cofactor for non-redox NAD^+^-dependent enzymes, declines with age, which is linked to multiple age-related diseases [130]. Blockade of de novo NAD^+^ synthesis triggers macrophage senescence by impairing the mitochondrial NAD^+^-dependent signaling and respiration [131]. In addition to T cells and macrophage, mitochondrial dysfunction also can induce microglial senescence in the CNS. In AD, mitochondrial dysfunction is associated with increased Aβ deposition, which results in premature senescence in microglia [132]. Taken together, mitochondrial dysfunction is a key mechanism underlying immunosenescence (Fig. 3).

### Cellular senescence

Cellular senescence is defined as an irreversible state of long-term cell cycle arrest caused by intrinsic or extrinsic stressors/damage. Senescent cells could trigger chronic inflammation by releasing SASP, which promotes cancer development and age-related tissue dysfunction [133]. Senescent non-immune cells (e.g., tumor cells) can drive immune cell senescence. For example, senescent cancer cells may upregulate PD-L1 or release extracellular vesicles containing PD-L1 to induce the senescence of T cells [134, 135]. As PD-L1 is ubiquitously upregulated in senescent non-immune cells during aging [136, 137], these cells may promote T cell senescence and functional decline via the PD-L1/PD-1 checkpoint signaling [134, 137–140]. Conversely, immune cell senescence has been demonstrated to propagate systemic cellular senescence, accelerating the aging of non-lymphoid organs [1], creating a pro-inflammatory, tumorigenic microenvironment [133, 141]. In the CNS, immune cell senescence intensifies neuroinflammation and cognitive deterioration during physiological brain aging [142]. In NDDs, microglia and astrocytes undergoing senescence enter irreversible cell cycle arrest and secrete a potent pro-inflammatory SASP, causing significant damage to neighboring neurons and accelerating neurodegeneration [143–145]. Collectively, cellular senescence in both immune and non-immune cells leads to the establishment of a vicious cycle, accelerating immune aging and age-related pathologies.

Currently, detection of senescent cells mainly relies on senescence hallmarks including cell cycle inhibition, DNA damage response, SASP, increased lysosomal content, nuclear reorganization, and enhanced anti-apoptotic pathways (Fig. 4a) [146, 147]. However, identifying senescent immune cells during aging is challenging due to the complexity of immune cell biology, heterogeneity of senescent immune cells, and the overlap of senescence markers with other cellular states [147]. Importantly, recent studies have identified several immune cell-specific senescence biomarkers, such as increased levels of CD57 [148, 149], killer cell lectin-like receptor G1 (KLRG1) [148, 149], NK group 2, member D (NKG2D) [150] and CD153 [151, 152], and a loss of CD28 [153, 154]. Therefore, altered cell surface signaling is a defining characteristic of immune cell senescence (Fig. 4a).

### Stem cell exhaustion

Numerous studies have demonstrated that several immunosenescence hallmarks, such as genomic instability [155–157], telomere attrition [81], epigenetic dysregulation [87, 88], chronic inflammation [158, 159], mitochondrial dysfunction [120–122] and loss of proteostasis [95, 97], contribute to HSC aging (Fig. 4b). For instance, diminished DNA DSB repair exacerbates HSC aging, depleting HSCs and impairing self-renewal [157], while reduced expression of mini-chromosome maintenance (MCM) helicase drives replication stress, causing cell cycle arrest and chromosomal instability in HSCs [160]. Chronic inflammation-induced cytokines contribute to HSC aging. Specifically, aging-related cytokines induce Id1 expression, impairing HSC self-renewal; conversely, Id1 deletion enhances self-renewal while reducing proliferation and mitochondrial biogenesis [161].

### Chronic inflammation

Chronic inflammation is a hallmark of immune aging (Fig. 5) [162]. It can be non-sterile anti-pathogen immune responses or sterile, low-grade inflammation, also known as inflammaging. According to previous literature, both chronic infection and inflammaging are closely associated with immunosenescence (Fig. 5) [79, 158, 163–165]. Chronic inflammation contributes to immunosenescence not only by weakening the self-renewal capacity of HSCs [158, 159, 166–169], but also by accelerating the senescence of immune cells (Fig. 5) [61, 163], leading to immune function decline and accumulation of senescent cells and inflammatory mediators [165]. For example, NLRP3 inflammasome activation causes age-related thymic atrophy and immunosenescence [163], while deletion of NLRP3 and ASC in inflammasome assembly rescues the age-related thymic demise, increases T cell progenitors, and alleviates immune senescence [163, 164]. The connection between NLRP3 inflammasome activation and broader systemic aging processes provides a valuable insight into immune aging (Fig. 5). In addition to NLRP3 inflammasomes, the cGAS-STING signaling pathway is also a key inducer of chronic inflammation and is linked to immune aging (Fig. 5) [61, 170]. In the CNS, mtDNA released from dysfunctional mitochondria activates cGAS in aged microglia, leading to STING activation, microglial aging, neurodegeneration, and cognitive impairment [61]. Apart from the cGAS-STING pathway, other pattern recognition receptors, such as Toll-like receptors, NOD-like receptors, and aryl hydrocarbon receptor, also can sense sterile triggers and induce chronic inflammation [162]. ADAR1 (adenosine deaminase acting on RNA 1) has been increasingly recognized for its broader role in modulating chronic inflammation [171], while its role in immune aging remains underexplored and warrants deeper investigation.

### Altered intercellular communication

BM microenvironment is a critical determinant for HSC homeostasis. Multiple stromal cells can regulate the BM niche to shape HSC aging (Fig. 6) [172–178]. Sympathetic innervation of the BM can also influence HSC aging, as loss of sympathetic nerves or adrenoreceptor β3 signaling triggers premature HSC aging in young mice. Strikingly, selective activation of β3 adrenoreceptor significantly rejuvenates the aged HSCs [179, 180]. In addition, immunosenescence is orchestrated by a complex network of intercellular molecular mediators. For instance, reduced IGF1 in the local BM microenvironment is a driver of HSC aging, while IGF1 treatment reverses several hallmarks of aging in aged HSCs [181]. Furthermore, recent studies on heterochronic parabiosis have revealed systemic distribution of pro-aging mediators in the blood. The heterochronic parabiosis induces a progressive immune decline in the young heterochronic partners [182]. Moreover, injection of aged plasma from 20-month-old mice into 5-month-old younger mice reduces the percentages of naïve T cells in the spleen and blood, and increases memory CD8^+^ T cells and monocytes in the spleen [183]. β2-Microglobulin (β2M) is a mediator of these pro-aging effects. Its level rises with age and correlates with increased circulating Ly6C^High^ monocytes [184].

Immune checkpoints, such as the PD-L1/PD-1 axis, enable senescent cells to escape CD8^+^T/NK cell-mediated immunosurveillance [136–138, 185, 186]. TIGIT (T-cell immunoreceptor with immunoglobulin and immunoreceptor tyrosine-based inhibitory motif domain) has been shown to be a novel co-inhibitory receptor, which is upregulated on CD8^+^ T cells with age [187]. Senescent CD8^+^ T cells also express high levels of senescence-associated inhibitory receptor KLRG1, which contributes to immune aging of CD8^+^ T cells [188]. Ganglioside GD3, recently identified as an immune checkpoint enabling senescent cell evasion of immunosurveillance [189], may similarly drive immune cell aging phenotypes.

### Microbiome dysbiosis

Older individuals exhibit age-related changes in the gut microbiome (Fig. 6) [190–192]. Aged microbiome generates pro-inflammatory cytokines (e.g., TNF-α, IL-2, IL-6, IL-8, IFN-γ) that increase systemic inflammation and exacerbate immune aging (Fig. 6) [192, 193]. Conversely, fecal microbiota transplantation (FMT) from young mice to aged mice rejuvenates the aged HSCs, promotes lymphoid differentiation and reduces myeloid differentiation, highlighting an essential role of microbiota in the regulation of HSC aging [194]. FMT from young mice also suppresses pro-inflammatory responses [194], activates the anti-aging FOXO signaling pathway [194], and rescues immune aging in brain and cognitive impairments in aged mice [195]. Furthermore, gut microbiota promotes neutrophil aging through Toll-like receptor and myeloid differentiation factor 88 pathways [43]. Depletion of microbiota significantly diminishes circulating aged neutrophils, and reduces inflammation-related organ damage [43]. During aging, microbiota induces aging-related alterations of gene expression in microglia [52], while depletion of gut microbiota attenuates oxidative stress and mitochondrial dysfunction in aged microglia [52]. Consistently, commensal bacteria drive the accumulation of senescent microglia and disease-associated microglia (DAM) during aging [196]. This connection between gut microbiome and microglial aging highlights the broader implications of intestinal health on neuroinflammation and neurodegeneration.

## Immunosenescence and brain aging

Immunosenescence occurs in both the peripheral and the central immune systems, and contributes to aging and age-related diseases. The aged peripheral and central immune cells may modulate brain structure and function, leading to brain aging and age-associated neurodegeneration. In this section, we will analyze the potential links and molecular mechanisms connecting immunosenescence with brain aging based on the 11 mechanistic hallmarks discussed above.

**Fig. 7:**
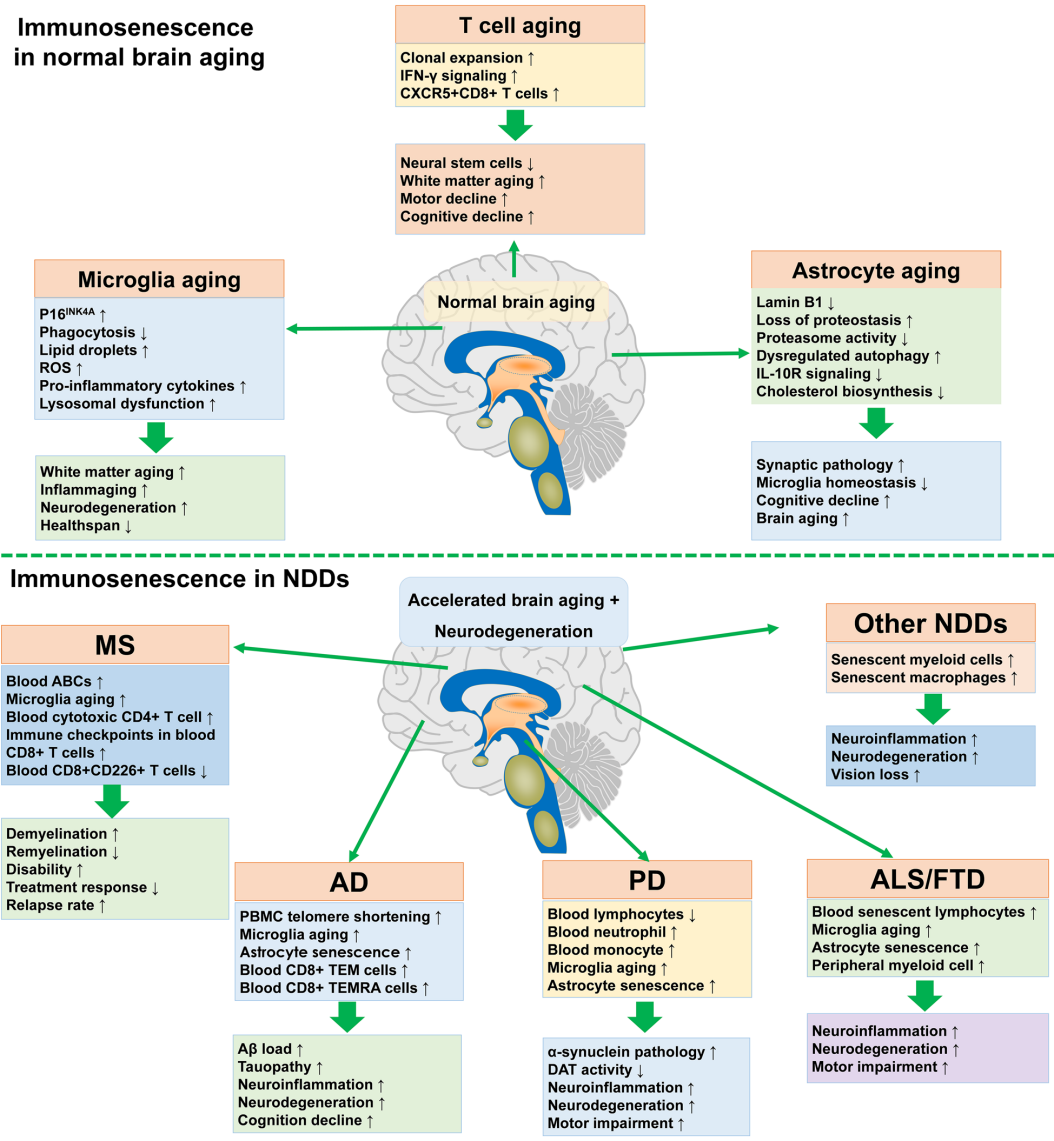
The potential role of immunosenescence in brain aging and NDDs. During normal brain aging, the aging of microglia, astrocytes, and T cells contributes to white matter degeneration, inflammaging, and age-dependent declines in both cognitive and motor function. These age-related immune changes exacerbate neuroinflammatory processes and disrupt neurovascular function, leading to deterioration of neural integrity. Furthermore, in NDDs, such as AD, PD, and ALS, immunosenescence is implicated in the initiation and acceleration of pathological protein aggregation, progressive neuronal loss, and subsequent behavioral and cognitive deficits. ROS: reactive oxygen species; CXCR5: C-X-C motif chemokine receptor 5; ABCs: age-associated B cells; NDD: neurodegenerative diseases; MS: multiple sclerosis; AD: Alzheimer’s disease; PD: Parkinson’s disease; ALS/FTD: amyotrophic lateral sclerosis/frontotemporal dementia; PBMC: peripheral blood mononuclear cells; TEM: effector memory T cells; TEMRA: effector memory cells re-expressing CD45RA; Aβ: β-amyloid; DAT: dopamine transporter

p16^INK4A^ is a well-established biomarker of cellular senescence. Talma et al. (2021) found that in 2-year-old mice, p16^High^ cells are significantly increased throughout the CNS and modulate key neuroimmune processes including inflammation and phagocytosis [197]. Most of the p16^High^ cells are microglia, which were also confirmed in aged human brain (Fig. 7). They further classified the p16^High^ microglia into two different subsets, one expressed across the lifespan and the other age-associated, revealing heterogeneity in microglial aging. Interestingly, these p16^High^ microglia subpopulations are phenotypically distinct from DAM and exhibit only partial expression of senescence markers [197], pointing to a unique state. The functional heterogeneity of aged microglia is further supported by work showing that aging induces distinct inflammatory programs across microglial subsets [198]. These divergent profiles imply specialized contributions to age-related neuroinflammation and NDDs. Li et al. (2023) delineated the transcriptional and epigenetic landscape of microglia from 3- to 24-month-old mice and identified age-dependent genes and differential ligand–receptor interactions in aged microglia, which are associated with myelination reduction and cognitive decline in old mice [199]. In humans, age-associated microglial genes are enriched in processes that show evolutionary conservation between mice and humans, including lipid metabolism, immune responses, axonal guidance, cell adhesion, and actin dynamics [200, 201]. These findings indicate that microglia undergo widespread age-dependent remodeling throughout the lifespan.

Microglial senescence accelerates brain aging, driving chronic inflammation and increasing susceptibility to NDDs (Fig. 7) [202]. Senescent microglia exhibit a distinct aging-associated gene signature, marked by elevated expressions of osteopontin and chitotriosidase 1, both elevating the risk of neurodegeneration [203, 204]. One of the most prominent markers of senescent microglia is the buildup of intracellular lipid droplets, observed extensively in both mice and humans (Fig. 7) [205]. These lipid-droplet-accumulating microglia exhibit several senescent features such as phagocytosis deficiency, excessive ROS production, and release of pro-inflammatory cytokines (Fig. 7) [205]. With advancing age, myelin pieces resulting from myelin fragmentations are gradually released into the brain parenchyma and engulfed by microglia [53]. This exacerbates the phagocytosis deficiency in aged microglia, leading to the formation of insoluble, lipofuscin-like lysosomal inclusions, resulting in lysosomal dysfunction, microglia senescence, and immunodeficiency [53].

The phagocytic activity of aged microglia is regulated by multiple molecular mechanisms, such as CD36 and CD22 signaling pathways. The scavenger receptor CD36 is a positive regulator of phagocytic activity in mouse and human microglia, which is decreased during aging [206]. The age-dependent impairment of phagocytic activity due to CD36 deficiency can be rescued by CD36 overexpression or vitamin B3 treatment [206]. Conversely, CD22, typically a canonical B cell receptor, negatively regulates phagocytosis in aged microglia by interacting with α2,6-linked sialic acid [207]. Mechanistically, the α2,6-linked sialic acid interacts with CD22 to suppress microglia phagocytosis during aging [207]. Importantly, blockade of CD22 reduces the abnormal accumulation of myelin debris, Aβ aggregates, and α-synuclein fibrils by rejuvenating aged microglia [207]. Collectively, CD36 and CD22 emerge as opposing regulators of microglial phagocytosis during aging. Future studies should define the therapeutic potential of targeting these pathways (e.g., CD36 augmentation, CD22 blockade) to mitigate pathological protein accumulation and preserve cognitive function in diverse aging populations.

The molecular and cellular phenotypes of senescent astrocytes have not yet been fully identified, and their physiological roles in the aging brain remain poorly understood. Lee et al. (2022) identified a unique senescent astrocyte subtype in the aging mouse hippocampus, which exhibits loss of proteostasis caused by reduced mTOR and proteasome activities with lysosomal dysfunction (Fig. 7) [60]. These senescent astrocytes display impaired secretion of synaptogenic molecules and reduced astrocytic synapse elimination, thereby contributing to impaired synaptic plasticity [60]. Lipopolysaccharide challenge can induce astrocyte senescence in aged hippocampus [208]. These senescent astrocytes exhibit defects in IL-10R signaling and cholesterol biosynthesis, thereby impairing microglia homeostasis during innate immune activation (Fig. 7) [208]. Similar to other cells, a loss of Lamin B1 is also a core feature of senescent astrocytes. The reduction of Lamin B1 occurs in aged hippocampal astrocytes across species, thereby leading to nuclear malformations and loss of nuclear circularity [209]. Understanding the role of senescent astrocytes in the aging brain is critical for developing interventions to preserve cognitive function and mitigate the progression of NDDs. Future investigations should aim to delineate the causal relationships between astrocyte senescence and specific brain aging-related pathologies.

Senescent T cells have been linked to cognitive decline, demonstrating associations with poor memory in patients with rheumatoid arthritis (Fig. 7) [210]. Mechanistically, accumulation of CD8^+^ T cells, potentially mediated by the CXCL10-CXCR3 signaling, drives axon degeneration and myelination impairment during normal aging, contributing to cognitive and motor decline [211, 212]. Moreover, clonally expanded aged CD8^+^ T cells in the CNS express high levels of IFN-γ, which reduces the proliferation of neural stem cells in vitro and in vivo [58]. Consistently, Kaya et al. (2022) demonstrated that CD8^+^ T cells induce oligodendrocyte loss through IFN-γ signaling during aging, contributing to white matter aging [213]. CD8^+^ T cells also impair axonal regeneration capacity during aging [6]. Mechanistically, lymphotoxin-induced CXCL13 recruits CXCR5^+^CD8^+^ T cells to injured axons, where they suppress regeneration through caspase-3 activation [214]. Collectively, these findings suggest that aged CD8^+^ T cells play an important role in mediating axon degeneration, myelination dysfunction, and cognitive decline during normal brain aging (Fig. 7). The involvement of senescent T cells in normal brain aging and age-dependent cognitive decline underscores the complexity of immune interactions within the aging brain. Further studies are needed to understand the full scope of their impact across different cognitive domains and neurological conditions.

**Fig. 8:**
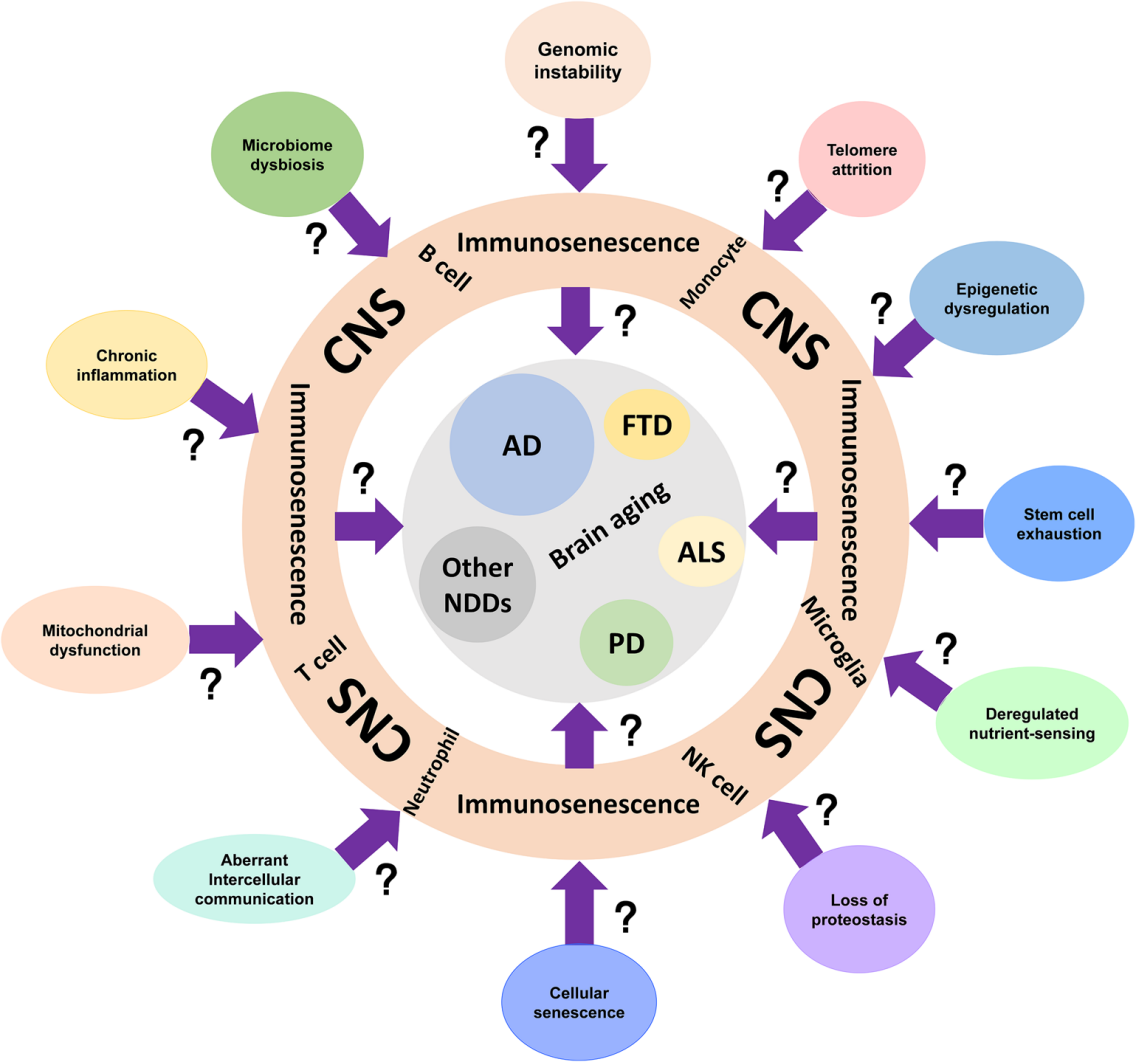
Research directions for brain aging and NDDs in light of immunosenescence hallmarks. The absence of dedicated mechanistic studies on immune cell aging within CNS creates a major gap in understanding how age-related declines in CNS immunosurveillance and homeostasis facilitate both brain aging and neurodegeneration. Closing this gap is vital, as determining if and how these core aging hallmarks cause CNS immune aging could reveal novel, cell-specific therapeutic targets within the brain’s immune system to stop or reverse brain aging and NDDs. This requires shifting focus beyond peripherally validated approaches. Future work must therefore directly and rigorously examine these hallmarks in microglia and related CNS immune cells in aging and NDDs

Despite compelling evidence implicating senescence of microglia and other CNS immune cells in brain aging, the fundamental molecular mechanisms and biological processes governing their aging phenotypes remain poorly defined. While our proposed framework of 11 mechanistic hallmarks provides a systematic scaffold for investigation, rigorous validation is needed to confirm their specific roles in initiating and sustaining immune cell senescence in the CNS (Fig. 8), and their causal link to neuroinflammation, neurodegeneration, and cognitive decline.

Although a systematic understanding of the mechanisms driving the senescence of microglia and other CNS-resident immune cells remains elusive, emerging data confirm that their aging phenotypes are multifactorially regulated. Progranulin (PGRN, encoded by *GRN*) is a key regulator of lipid droplet formation in microglia [205]. *GRN* deficiency induces a series of senescent phenotypes in human induced pluripotent stem cell (hiPSC)-derived microglia, such as oxidative stress, lysosomal dysfunction, and endosome damage, which can be reversed by *GRN* replacement therapy [215]. The ATP–P2X_7_R signaling pathway also regulates microglial senescence, as pathological activation of ATP–P2X_7_R signaling accelerates microglial senescence by suppressing PINK1 (PTEN-induced kinase 1)-mediated mitophagy [216]. Rapamycin-insensitive companion of mTOR (RICTOR) is a core component of the mTORC2 complex [106], which is linked to ageing and development. Aged microglia exhibit decreased expression of RICTOR, significantly contributing to aging-associated phenotypes in microglia [217]. Cellular senescence in astrocytes can be induced by various intrinsic and extrinsic factors, including oxidative stress, proteasome inhibition, and replicative exhaustion, all of which contribute to normal brain aging [218]. However, the molecular mechanisms orchestrating astrocyte senescence remain largely uncharacterized. Activator protein-1 (AP-1) is a heterodimeric transcription factor composed of Jun, Fos, Maf and activating transcription factors (ATFs) [219]. AP-1 drives cellular senescence in glia of CNS in flies [220–222]; however, whether AP-1 also regulates glia cell senescence in mice or humans remains unknown, and deserves further explorations.

Previous research has established that brain can influence a variety of peripheral immune profiles, including T cells, B cells, and other immune cell types [223, 224]; however, whether normal brain aging contributes to immune system aging remains largely unexplored. Future research should focus on delineating the precise pathways by which age-related changes in the brain impact systemic immune aging, which could provide crucial insights into age-related disorders and open new therapeutic avenues for both neurodegeneration and immune dysfunction.

## Immunosenescence and NDDs

As mentioned above, aging in both the peripheral immune system and the CNS immune system is closely linked to brain aging. In NDDs, aged immune cells in both periphery and CNS may also regulate the neurodegenerative processes, thereby influencing disease progression and prognosis of patients. In the following sections, we will analyze the potential connections between immunosenescence and NDDs as well as the molecular mechanisms.

### Immunosenescence in MS

Immunosenescence occurs in experimental autoimmune encephalomyelitis (EAE) animal models [13] and MS patients [13, 196, 225]. Immune aging has been linked to increased disability, reduced therapeutic response, higher relapse rate, and elevated conversion risk from relapsing to progressive MS [11]. Aged MS patients exhibit a specific pattern of immunosenescence, featured by aberrant activation of CD4^+^ T cells and increases of cytotoxic CD4^+^ T cells in the blood [12]. By contrast, CD8^+^ T cells in older MS patients express multiple immune checkpoint molecules, such as KLRG1, LAG3, and CTLA-4 [225], along with decreased expression of costimulatory molecule CD226, suggesting premature aging in CD8^+^ T cells [225]. Compared to other types of immune cells, peripheral B cells in MS patients display accelerated epigenetic age, indicating heterogeneity of immune cell aging in MS patients [226]. Specifically, IgD-CD27^−^ and CD21^−^CD11c^+^ B cells, identified as ABCs with pro-inflammatory profiles, are elevated in the peripheral blood of MS patients compared to age-matched healthy controls, indicating a pro-inflammatory role of aged B cells in the autoimmune response of MS [227]. Additionally, these B cell subtypes are significantly more abundant in the CSF than in the peripheral blood, demonstrating compartmentalized B cell aging in MS [227]. Notably, in a subgroup of MS patients with absence of lipid-specific oligoclonal IgM bands, aging induced remarkable decreases of T cells and B cells in the CSF, suggesting premature immune aging in these individuals [228]. Moreover, aging in MS is also linked with increased PD-L1 and activin A, two markers of immune aging, in the CSF [228]. Furthermore, microglia in the white matter undergo accelerated cellular senescence in the brain and spinal cord of EAE models [196], while knockout of p16^INK4A^ alleviates spinal cord demyelination occurring in EAE [196]. This result underscores a key role of microglial senescence in the pathogenesis of MS. Additionally, the senescence-associated phenotypes in microglia persist within demyelinated lesions during aging, contributing to inefficient remyelination and progressive myelin damage [229]. Mechanistically, aged microglia may drive chronic neuroinflammation and MS progression through impaired phagocytic capacity and increased production of inflammatory factors [230]. Collectively, these findings suggest that immune aging may contribute to progressive myelin impairment in MS (Fig. 7).

Paradoxically, a recent study found that neither the senolytic cocktail dasatinib + quercetin (DQ) nor genetic clearance using *INK-ATTAC* transgenic mice ameliorated EAE severity, implying limited therapeutic potential of senolytics in MS [231]. While the association between microglial senescence and MS progression is well-documented, significant knowledge gaps remain regarding: (1) the precise molecular cascades linking senescence to demyelination; (2) mechanisms underlying the limited efficacy of senolytics; and (3) alternative therapeutic strategies beyond senolytic approaches. Future studies are needed to reveal mechanistic dissection of immune cell-specific aging pathways and develop interventions targeting the senescence–neuroinflammation–demyelination axis.

### Immunosenescence in AD

The close association between immunosenescence and AD has been increasingly recognized (Fig. 7) [232]. AD patients display accelerated immunosenescence compared to age-matched control individuals. For example, peripheral blood mononuclear cells from AD patients exhibit significant telomere shortening compared to those from control individuals [233]. Consistently, shorter telomere length in blood leukocytes is an independent risk factor for AD [234]. Specifically, the telomere length in T cells, but not that in B cells or monocytes, is positively associated with global cognition measured by Mini-Mental Status Examination scores [233]. Young et al. (2023) reported that AD patients exhibit an age-associated increase of CD8^+^ TEM cells that express low levels of IL-7 receptor alpha (IL-7Rα^low^) [235]. They further identified a specific gene signature representing the association between normal aging and IL-7Rα^low^ CD8^+^ TEM cells [235]. Importantly, these IL-7Rα^low^ aging genes are associated with cognitive function as assessed by Montreal Cognitive Assessment [235]. AD also has an immune signature consisting of CD8^+^ TEM cells expressing CD45RA (TEMRA), which are considered senescent and negatively associated with cognitive performance [236]. These results suggest that immune aging in peripheral blood is associated with disease progression of AD, underscoring the potential of immune aging markers in blood as biomarkers for predicting disease progression and cognitive decline.

Microglia also undergo significant premature aging in AD (Fig. 7). Senescent microglia have been identified in the brains of mouse models and patients with tauopathies [237–239]. Tau exposure causes cell cycle arrest, loss of Lamin B1, altered H3K9me3 histone modification, and production of SASP, all being canonical markers of senescence [237]. Similarly, Aβ also accelerates senescence of human microglial cells, characterized by upregulation of p21 and SA-β-gal [240]. Importantly, p16^INK4A^-expressing senescent microglia are specifically localized near amyloid plaques in the brains of AD patients and 5 × FAD mice [241]. Consistently, Fancy et al. (2024) also identified enhanced premature microglial senescence in postmortem brains from AD patients [132]. The senescent microglia exhibit reduced phagocytic capacity, resulting in greater Aβ load [132]. Mutations in TREM2 confer a higher risk of AD [242]. Rachmian et al. (2024) identified a specific type of senescent microglia expressing high levels of TREM2, which exhibit a distinct signature from TREM2-dependent DAM [9]. Ferroptosis is a pathological marker of senescent microglia [243]. Senescent microglia exhibit extensive lipid peroxidation, accumulation of lipid droplets, enhanced expression of phospho-H2AX, and differential expression of ferroptosis-related genes involved in iron-mediated lipid dysmetabolism and oxidative stress [243]. Even in patients with Down syndrome who usually progress to AD dementia in late stage of the disease, microglia also undergo cellular senescence characterized by elevated type I IFN signaling [244]. Similar to other immune cells, sustained proliferation of microglia also promotes their replicative senescence, characterized by increased SA-β-gal and telomere shortening, which are associated with the development of DAM [245]. Wei et al. (2023) found that senescent microglia exhibit increased H3K18 lactylation and pan-histone lysine lactylation in naturally ageing mice and AD mice, thereby accelerating brain aging and AD neurodegeneration through the NF-κB signaling pathway [246]. Together, premature senescence of microglia plays a pivotal role in AD pathogenesis, which represents a potential therapeutic target for slowing or reversing AD progression.

Astrocyte senescence is another pathological feature of AD (Fig. 7). Astrocyte senescence induces excitotoxicity in cortical neurons, thereby contributing to neurodegeneration in AD [247]. Tau oligomers can induce astrocyte senescence by releasing HMGB1 and SASP, which drive paracrine senescence of adjacent cells [248]. Beyond tauopathy, Aβ, H_2_O_2_ and IL-1β also can induce astrocyte senescence [249]. Additionally, inactivation of YAP (Yes-associated protein) contributes to premature astrocyte senescence in the hippocampus of aging and AD mice, characterized by cell proliferation inhibition, increased SA-β-gal activity, reduced Lamin B1, and upregulation of SASP [250]. The induction of senescence in astrocytes highlights a complex interplay between cellular aging and neurodegenerative processes in AD; however, the direct causality warrants further investigation.

Rejuvenating immune cells has shown promise in alleviating AD-associated phenotypes, providing potential therapeutic avenues. For example, p16^INK4A^ knockdown in BV2 microglia enhances Aβ phagocytosis through regulation of the cell cycle [241]. In *MAPT*^P301S^ PS19 mouse model of tauopathy, clearance of p16^INK4A^-positive senescent astrocytes and microglia by using *INK-ATTAC* transgenic mice attenuates gliosis, tau hyperphosphorylation, and neurodegeneration [239]. Consistently, removal of senescent microglia with the senolytic BCL2 family inhibitor ABT-737 improves cognition and reduces brain inflammation in 5 × FAD mice [9]. In addition, transplantation of young immune cells into aged mice also alleviates AD-like phenotypes. For example, transplantation of young BM in aged APP/PS1 mice reduces Aβ plaque burden, neuroinflammation and neuronal degeneration, and improves behavioral deficits [16], highlighting a critical role of immune aging of BM in AD pathogenesis. Additionally, transplantation of young WT splenocytes promotes Aβ clearance, reduces astrogliosis, increases systemic level of growth differentiation factor 11, and improves the spatial learning and memory of APP/PS1 transgenic mice [251], further implicating the key role of peripheral immune aging in AD. Collectively, these findings highlight the potential of targeting immunosenescence, particularly microglial aging, as a promising therapeutic strategy for AD (Fig. 7).

### Immunosenescence in PD

Inflammation and immune dysfunction are increasingly implicated in both initiation and exacerbation of neurodegeneration in PD [252–254]. However, the role of immunosenescence in PD pathogenesis remains less understood. Accumulating evidence highlights significant aging-associated changes in the peripheral immune system of PD patients (Fig. 7) [34, 255]. Both peripheral total T and B cell counts decrease with normal aging and exhibit further reductions in PD patients [34]. PD patients also display decreased naïve CD4^+^ and CD8^+^ T cells [255, 256], which are associated with autonomic dysfunction and psychiatric complications [255]. Additionally, naïve B cells, NK cells, and plasma cells are also reduced in PD patients compared to healthy controls [257, 258]. Interestingly, lower lymphocyte count in normal aging has been revealed to be associated with increased risk of PD in longitudinal cohort studies, indicating that aging-related lymphocyte decline may increase PD susceptibility [15, 259]. Consistently, lower lymphocyte counts correlate with reduced striatal dopamine transporter levels, highlighting their potential link to dopaminergic degeneration [260]. In contrast, PD patients exhibit increased late-differentiated senescent CD4^+^ and CD8^+^ T cells, which are negatively correlated with the age of disease onset [255]. Moreover, PD patients also display higher neutrophil and monocyte levels, an indication of myeloid-biased differentiation, which is a typical marker of immunosenescence [261]. The increased blood neutrophil counts in PD patients are associated with higher motor impairment and greater reductions of striatal dopamine transporter activity in the caudate and putamen [261]. Interestingly, patients with isolated rapid eye movement sleep behavior disorder also exhibit premature immune aging in peripheral blood, characterized by decreases in absolute counts of CD3^+^ T cells, increased frequency of CD8^+^ TEM cells, and reduced memory B cells [262], indicating that immunosenescence occurs even in prodromal PD patients. Controversially, some studies revealed that PD patients exhibit an atypical CD8^+^ T cell senescence pattern featured by reduced expression of p16^INK4A^, indicating a lack of the CD8^+^ T cell replicative senescence that characterizes normal ageing [263, 264]. Overall, the consistent observation of peripheral immune aging in PD highlights its potential role in the pathogenesis of PD, yet the mechanisms remain to be fully elucidated. The apparent contradiction in T cell senescence patterns raises important questions about the heterogeneity of immune aging in PD and its implications for disease progression (Fig. 7). Longitudinal studies tracking immune aging markers with disease progression could provide valuable insights into the temporal relationship and causative role of peripheral immune alterations in PD.

Reduction of phagocytosis capacity is a typical feature of aged microglia/monocytes. Following intra-striatal injection of human α-synuclein preformed fibrils (PFFs), microglia in aged mice show greater accumulation of α-synuclein aggregates than microglia in younger mice, due to the diminished clearance capacity of the autophagy-lysosome system [265]. Moreover, microglia in adult mice exhibit significant defects in the phagocytosis of free and extracellular vesicle-associated α-synuclein oligomers compared to microglia in young mice [10]. Additionally, monocytes from elderly individuals also display reduced phagocytosis of extracellular α-synuclein [10]. The observed deficits in microglial and monocyte clearance underscore the role of aged immune cells in PD pathology. However, the specific molecular mechanisms require further investigation (Fig. 7).

α-Synuclein pathology is a key trigger of microglial senescence, which in turn exacerbates the pathological aggregation of α-synuclein. Selective overexpression of α-synuclein-A53T in the substantia nigra pars compacta induces pro-inflammatory SASP, expression of senescence-related protein markers (SA-β-gal, p16, p21, and γ-H2AX), as well as mitochondrial dysfunction [266], indicating that α-synuclein pathology triggers premature aging in the CNS. Interestingly, α-synuclein-containing small extracellular vesicles derived from PD models induce increased PD-L1 expression in both CD4^+^ and CD8^+^ T cells, which mediates the suppression of CD4^+^ and CD8^+^ T cells [139]. Moreover, α-synuclein PFFs induce a loss of Lamin B1 and an increase of p21 in reactive microglia in mouse brains [267]. This provides evidence for the role of α-synuclein pathology in accelerating senescence processes, particularly within immune cells, which may contribute to the progression of PD. However, the mechanistic links between α-synuclein-induced senescence and clinical manifestations of PD still require further clarification.

Astrocytes also undergo senescence during aging, resulting in abnormal activation of BV2 microglia and death of N2a cells [268]. In PD, α-synuclein pathology is associated with astrocyte senescence characterized by cell cycle arrest, nuclear lamina deficiency and presence of senescence-associated heterochromatin foci [269]. In addition, neurotoxins such as pesticide rotenone and paraquat, also cause astrocyte senescence [270, 271]. The senescence of astrocyte exaggerates the degeneration of midbrain neurons derived from hiPSCs of PD patients with *SNCA* gene duplication [270], while depletion of senescent cells protects against paraquat-induced neuropathology [271]. Like in other senescent cells, the cGAS/STING pathway is upregulated in senescent astrocytes in PD models. Interestingly, selective depletion of cGAS in astrocytes significantly reduces senescence hallmarks and neurodegeneration in a PD mouse model [272], emphasizing an essential role of the cGAS/STING pathway in astrocyte senescence. Taken together, astrocyte senescence is evident in multiple PD models and contributes to the progressive neurodegeneration observed in PD. These findings suggest that targeting immune aging, particularly the senescence of microglia and astrocytes, could be a promising therapeutic strategy for slowing PD progression (Fig. 7).

### Immunosenescence in amyotrophic lateral sclerosis (ALS) and frontotemporal dementia (FTD)

Immune aging may also play a potential role in the pathogenesis of ALS and FTD (Fig. 7). Yildiz et al. (2022) showed significant elevations of senescent and late memory T cells and B cells in the peripheral blood of ALS patients [273], indicating premature aging of peripheral immune cells in these individuals. Zaccai et al. (2024) showed that CD4^+^ T cell aging exacerbates neuroinflammation in a mouse model of ALS [274]. Beyond peripheral immune aging, numerous cells in the lumbar spinal cord of symptomatic *SOD1*^G93A^ rats exhibit increased p16^INK4A^ expression and a loss of Lamin B1, two typical markers of senescence. Many of these cells are Iba1^+^ microglia, suggesting the presence of immune aging in the CNS of *SOD1*^G93A^ rats. In vitro studies of microglia from *SOD1*^G93A^ rats revealed features of senescence, including elevated SA-β-gal activity, as well as increased expression of p16^INK4A^, p53, and matrix metalloproteinase-1 [275]. Astrocyte senescence, marked by increased expression of p16 and p21, has also been observed in the frontal cortex of ALS patients, thereby indicating a potential role of astrocyte senescence in ALS pathogenesis [276]. The most common genetic cause of ALS and FTD is the hexanucleotide repeat (GGGGCC) expansion in the noncoding region of the *C9orf72* gene. *C9orf72* is highly expressed in immune cells, especially in myeloid cells. *C9orf72* deficiency can drive lysosomal accumulation and age-related neuroinflammation in macrophages and microglia [277], highlighting a potential role of *C9orf72* in the regulation of immunosenescence in ALS/FTD. However, the mechanistic pathways linking immune cell senescence to neurodegeneration in ALS/FTD are not fully elucidated. It is unclear whether targeting immune senescence can effectively slow or halt disease progression, and if so, which immune cells should be prioritized for therapeutic intervention. In addition, animal models such as *SOD1*^G93A^ rats do not entirely recapitulate human disease, emphasizing the need for translational studies in ALS/FTD patients.

### Immunosenescence in other NDDs

Age-related macular degeneration (AMD) is one of the leading causes of vision loss in elderly population. It has been shown that NAD^+^ depletion induces the senescence of macrophage, leading to subretinal lipid deposition and neurodegeneration in AMD (Fig. 7) [278]. Notably, NAD⁺ supplementation reverses this cellular senescence and prevents AMD phenotypes, and senolytic therapies demonstrate protective effects against AMD-associated neurodegeneration [278]. These findings point to the potential of targeting senescent immune cells in the treatment of AMD. Additionally, subretinal injection of healthy macrophages promotes the clearance of senescent macrophages, reducing the burden of AMD [278]. These findings collectively suggest that the AMD-associated neurodegeneration may not stem from inflammatory injury, but from tissue repair deficiency due to immunosenescence—a paradigm shift from traditional views emphasizing chronic inflammation and oxidative stress [279]. However, whether immunosenescence is a primary driver or a secondary outcome of other pathological processes remains unclear, and further mechanistic studies are needed to untangle the complex interactions between aging, immune function, and retinal degeneration.

Langerhans cell histiocytosis is an inflammatory disorder caused by *MAPK*-activating mutations, which drive the differentiation of hematopoietic progenitors into senescent myeloid cells and the infiltration of senescent CD11a^+^ macrophages into brain parenchyma (Fig. 7) [280]. Patients with Langerhans cell histiocytosis subsequently develop progressive and incurable neurodegeneration [280]. Suppressing MAPK activity and associated senescence processes alleviates peripheral inflammation, reduces the infiltration of senescent macrophages, and improves neurodegeneration in these patients, suggesting that targeting senescence pathways may be a viable strategy in diseases beyond AMD [280].

### Pathogenesis of NDDs in light of immunosenescence hallmarks

Immune senescence is a pervasive feature across NDDs. Nevertheless, fundamental questions regarding how immune senescence specifically manifests within the CNS and contributes to NDD pathogenesis remain unresolved (Fig. 8). It is unclear whether the immune cells in the CNS undergo the same molecular cascades of immunosenescence as those implicated in peripheral immunosenescence, or if there are tissue-specific molecular cascades. For instance, do microglia experience telomere shortening similarly to circulating monocytes? Is mitochondrial dysfunction in aged microglia governed by the same principles observed in splenic macrophages? Does epigenetic dysregulation in CNS immune cells drive NDD-relevant inflammatory phenotypes distinct from those in the periphery? Bridging this gap is essential for elucidating whether and how these canonical hallmarks drive CNS immune senescence and unveiling novel, cell-type-specific therapeutic targets to halt or reverse NDD progression.

### The effects of NDDs on immune aging

In the CNS, aged microglia and astrocytes significantly contribute to aberrant accumulation of pathological proteins, such as Aβ and α-synuclein, which are hallmark features of NDDs. Conversely, the presence of these pathological proteins accelerates the aging process of microglia and astrocytes, establishing a vicious cycle that exacerbates neurodegeneration. As noted, peripheral immunosenescence is a contributor to NDDs, but whether NDDs reciprocally accelerate peripheral immune aging remains poorly understood. Existing literature suggests that pathological proteins implicated in NDDs, such as Aβ [281, 282], tau [283], and α-synuclein [284], all affect peripheral immune cells. For example, tau and α-synuclein both induce specific T cell response [283, 284]. However, whether these pathological proteins directly contribute to peripheral immune aging is still largely unknown. Longitudinal studies using advanced immunoprofiling and bioinformatics approaches may provide valuable insights.

## Therapeutic implications

**Fig. 9:**
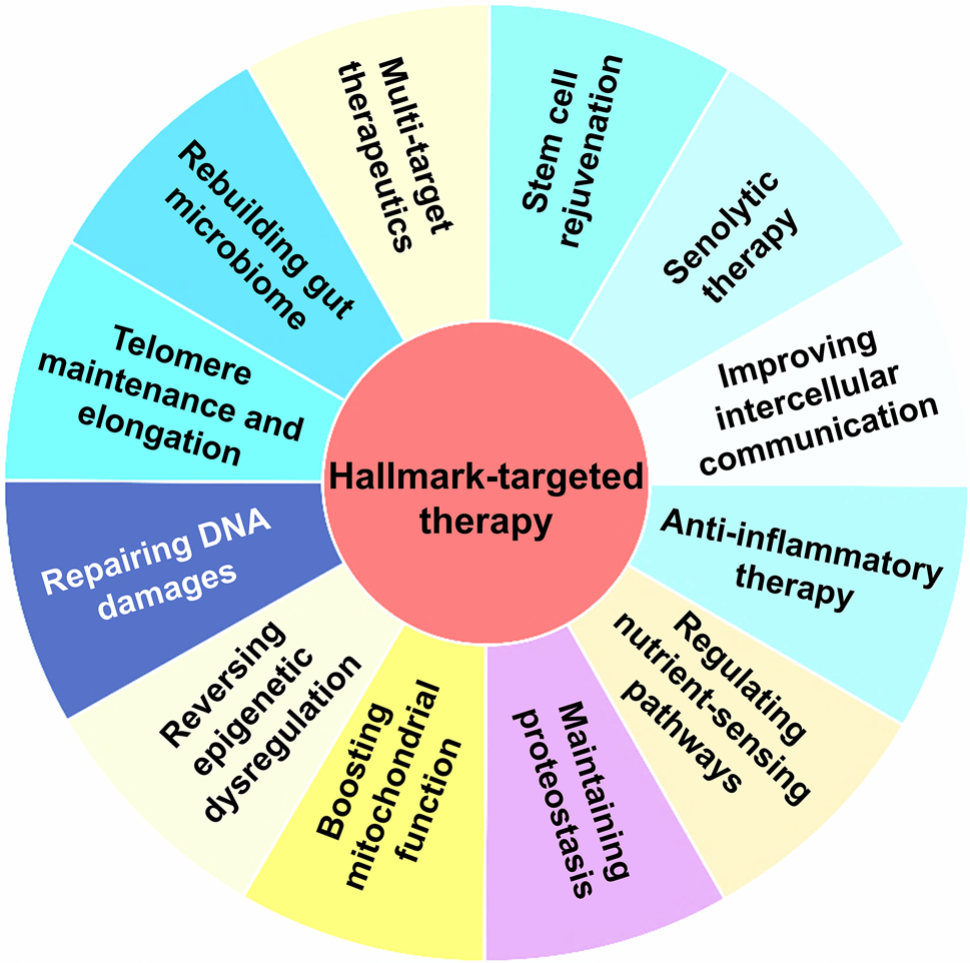
Hallmark-targeting therapies to revert immune aging and NDDs. Given that immunosenescence plays a key role in both normal aging and NDDs, we propose several hallmark-targeting therapies that may be beneficial for the treatment of NDDs by rejuvenating immune aging. As far as we are concerned, immunosenescence can be improved or even reversed by multiple hallmark-targeting interventions, including stem cell rejuvenation, senolytic therapy, improving intercellular communication, anti-inflammatory therapy, regulating nutrient-sensing pathways, maintaining proteostasis, boosting mitochondrial function, reversing epigenetic dysregulation, repairing DNA damages, telomere maintenance and elongation, rebuilding gut microbiome, and multi-target therapeutics

This section outlines hallmark-targeting strategies to rejuvenate the aged immune system, thereby combating brain aging and NDDs. Our discussion is organized around the 11 mechanistic hallmarks of immunosenescence (Fig. 9). Tables 1 and 2 respectively display evidence-based interventions for immunosenescence and NDDs, reflecting their developmental trajectory from theoretical speculation to preclinical and clinical stages.

**Table 1:** Evidence-based grading of 12 hallmark-targeting therapies for immunosenescence

Hallmark-targeting therapy	Theoretical speculation	Tested in preclinical stage	Tested in clinical stage
Stem cell rejuvenation	✓	✓	Not tested
Senolytic therapy	✓	✓	Not tested
Improving intercellular communication	✓	✓	Not tested
Anti-inflammatory therapy	✓	✓	Not tested
Targeting nutrient-sensing pathways	✓	✓	mTOR inhibitor RTB101: Phase 2b trial (ANZCTR, ACTRN12617000468325, NCT03373903) + Phase 3 trial (ANZCTR, ACTRN12619000628145) [320]
			mTOR inhibitor BEZ235 + RAD001: Phase 2a trial (ACTRN12613001351707)[321]
			mTOR inhibitor RAD001: Clinical trial (Trial registration number not available)[319]
			mTOR inhibitor rapamycin: Phase 2 trial (NCT02874924)[420]
			Calorie restriction: Phase 2 trial (NCT00427193)[421]
Maintaining proteostasis	✓	✓	Not tested
Boosting mitochondrial function	✓	✓	Not tested
Reversing epigenetic dysregulation	✓	✓	Not tested
Repairing DNA damage	✓	✓	Not tested
Telomere maintenance and elongation	✓	✓	Not tested
Rebuilding gut microbiome	✓	✓	Probiotics: Clinical trial (Trial registration number not available)[378]
Multi-target therapeutics	✓	✓	Moderate-vigorous physical activity: Clinical trial (ChiCTR2000039033)[401]
			Low-dose combined resistance and endurance training: Clinical trial (Trial registration number not available)[422]
			Strength endurance training: Clinical trial (Trial registration number not available) [423]
			BCG (Bacillus Calmette–Guérin) vaccine: Phase 4 clinical trial (NCT02953327) [424]

**Table 2:** Evidence-based grading of 12 hallmark-targeting therapies for NDDs

Hallmark-targeting therapy	Theoretical speculation	Tested in preclinical stage	Tested in clinical stage
Stem cell rejuvenation	✓	✓	Not tested
Senolytic therapy	✓	✓	DQ: Phase 1 trial in AD (NCT04063124)[294] DQ: Phase 2 clinical trial in AD (NCT04685590), ongoing
Improving intercellular communication	✓	✓	Anti-PD-L1 antibody IBCAb002: Ongoing phase 1 trial in AD (NCT05551741) Young fresh frozen plasma: Phase 1 tiral in AD (NCT05551741)[305] Plasma infusion: Phase 2 clinical trial in AD (NCT05068830), ongoing
Anti-inflammatory therapy	✓	✓	TNF-α blocker Etanercept: Phase 2 trial in AD (NCT01068353)[308]
Targeting nutrient-sensing pathways	✓	✓	mTOR inhibitor rapamycin: Phase 2 trial in ALS (NCT03359538)[325] Rapamycin: Phase 2 clinical trial in AD (NCT04629495), ongoing 12-week intermittent calorie restriction: Pilot trial in MS (NCT03539094)[425] Calorie restriction: Clinical trial in MS (NCT05327322), ongoing Time Restricted Eating: Phase 1 clinical trial in AD (NCT06548191), ongoing Ketogenic diet: Clinical trial in AD (NCT04701957), ongoing Ketogenic diet: Clinical trial in AD (NCT06681948), ongoing Ketogenic diet: Clinical trial in aMCI (NCT06444568), ongoing Ketogenic diet: Clinical trial in ALS (NCT04820478), ongoing Ketogenic diet: Pilot trial in PD (ACTRN12617000027314)[426] Ketogenic diet: Phase 2 trial in PD (NCT04584346)[427] Ketogenic diet: Phase 2 trial in AD (ACTRN12618001450202) [343] Ketogenic diet: Phase 2 trial in MS (NCT03718247) [344, 345] Ketogenic diet: Clinical trial in MS (NCT06715436), ongoing
Maintaining proteostasis	✓	✓	Spermidine supplementation: Phase 2a trial in old adults (NCT02755246) [346] Spermidine supplementation: Phase 2 trial in SCD (NCT03094546) [347]
Boosting mitochondrial function	✓	✓	NAD replenishment: Phase 1 trial in PD (NCT03816020) [327] NAD replenishment: Phase 1 trial in PD (NCT05344404) [326] Combined metabolic activators: Phase 2 trial in AD (NCT04044131) [330] Combined metabolic activators: Phase 2 trial in SCD and MCI (NCT04078178) [328] NAD replenishment: Clinial trial in MCI (NCT05344404) [329] NAD replenishment: Phase 2 trial in PSP, CBS, MSA (NCT06162013), ongoing NAD replenishment: Phase 2 trial in MCI and AD (NCT04430517), ongoing Sirtuin-NAD Activator MIB-626: Phase 1/2 trial in AD (NCT05040321), ongoing
Reversing epigenetic dysregulation	✓	✓	Resveratrol: Phase 2 trial in AD (NCT01504854) [363]
Repairing DNA damage	✓	Not tested	Not tested
Telomere maintenance and elongation	✓	✓	Not tested
Rebuilding gut microbiome	✓	✓	Fecal microbiota transplantation: Phase 2 trial in ALS (ChiCTR 2200064504) [381] Fecal microbiota transplantation: Phase 2 trial in PD (NCT04854291) [380] Fecal microbiota transplantation: Phase 2 trial in PD (ChiCTR1900021405) Fecal microbiota transplantation: Clinical trial in PD (NCT04837313), ongoing Fecal microbiota transplantation: Clinical trial in PD (NCT06388863), ongoing
Multi-target therapeutics	✓	✓	Metformin: Phase 2 trial in MCI (NCT01965756) [393, 394] Metformin: Phase 2 trial in MCI (NCT00620191) [392] Metformin: Phase 2 trial in MS (NCT05298670) [428] Metformin: Phase 2 trial in PD (NCT05781711), ongoing Metformin: Phase 1 trial in MS (NCT05349474), ongoing Physical exercise: Clinial trial in MS (NCT03679468) [429] Physical exercise: Clinial trial in MS (NCT06298201), ongoing Physical exercise: Clinial trial in MS (NCT05496881), ongoing Physical exercise: Clinial trial in MS (NCT06583967), ongoing Physical exercise: Clinial trial in MS (NCT06478784), ongoing Physical exercise: Clinial trial in MS (NCT05367947) [430] Physical exercise: Clinial trial in ALS (NCT03201991) [414] Physical exercise: Clinial trial in ALS (NTR1616) [431] Physical exercise: Clinial trial in ALS (Not available) [413] Physical exercise: Clinial trial in PD (RBR-83VB6B) [406] Physical exercise: Clinial trial trial in PD (NCT02593955) [405] Physical exercise: Clinial trial trial in PD (NTR4743) [404] Physical exercise: Phase 2 trial in PD (NCT01439022) [403] Physical exercise: Clinial trial trial in PD (NCT06324422), ongoing Physical exercise: Clinial trial trial in PD (NCT05815524), ongoing Physical exercise: Clinial trial trial in PD (NCT05854524), ongoing Physical exercise: Clinial trial trial in PD (NCT05720468), ongoing Physical exercise: Phase 2 trial in MCI (NCT02814526) [409, 410] Physical exercise: Phase 3 trial in AD (NCT01681602) [408] Physical exercise: Clinical trial in AD (NCT03890861), ongoing Physical exercise: Clinical trial in AD (NCT04956549), ongoing Physical exercise: Clinical trial in MCI (NCT05948930), ongoing BCG vaccine: Phase 2/3 trial in MS (NCT00202410) [432]

*AD* Alzheimer’s disease, *ALS* Amyotrophic lateral sclerosis, *BCG* Bacillus Calmette–Guérin, *CBS* Corticobasal syndrome, *DQ* Dasatinib + Quercetin *PD* Parkinson’s disease, *PSP* Progressive supranuclear palsy, *MCI* Mild cognitive impairment, *MS* Multiple sclerosis, *MSA* Multiple system atrophy, *NAD* Nicotinamide adenine dinucleotide, *SCD* Subjective cognitive decline

### Stem cell rejuvenation

As mentioned earlier, HSC aging, driven by both intrinsic defects and non-autonomous mechanisms, contributes significantly to HSC exhaustion and declined immune function. Therefore, therapies targeting either intrinsic or extrinsic triggers hold promise for rejuvenating HSCs and potentially reversing aging-associated immune phenotypes. Recent studies have identified several therapeutic targets, including Phf6 [89], MCM DNA helicase [255], mTOR signaling [113], Id1 [161], and cGAS/STING signaling [285]. In addition to targeting intrinsic factors, rehabilitating HSC niche represents a promising approach to further rejuvenating HSCs. Key therapeutic targets for BM niche repair include adrenoreceptor β3 signaling [179, 180], IGF1 [181], PIEZO1 [174], β2M [184], IL-1β [166] and adiponectin receptor signaling [168]. By restoring the supportive microenvironment, these therapies aim to enhance the regenerative potential of HSCs, improving their capacity to maintain hematopoiesis and immune function during aging. In NDDs, evidence has shown that transplantation of young BM diminishes Aβ plaque burden, neuroinflammation and neuronal degeneration, and improves behavioral deficits in aged APP/PS1 mice [16], highlighting the promise of young BM transplantation for NDD treatment. While targeting HSC aging presents a compelling strategy for rejuvenating the aged immune system, the extent to which this approach can yield systemic or neurological benefits remains unclear. Future research should explore whether reversing HSC aging can impact neurodegenerative processes, such as those seen in AD and PD.

### Senolytic therapy

Cellular senescence is a key hallmark of immune aging, thereby acting as a promising therapeutic target for anti-aging interventions. Baker et al. (2011) designed a transgene, *INK-ATTAC*, for selectively eliminating p16^INK4A^-positive senescent cells upon AP20187 administration. Interestingly, this approach successfully alleviated the progression of age-related phenotypes in the BubR1 progeroid mouse model [286]. ABT263 is a potent senolytic drug that selectively inhibits anti-apoptotic proteins BCL-2 and BCL-xL, effectively eliminating senescent cells in normal aged mice [287]. Strikingly, removal of senescent cells with ABT263 rejuvenated not only HSCs from a model of irradiation-induced premature aging but also those from physiologically aged mice [287]. Amor et al. (2020) identified the urokinase-type plasminogen activator receptor (uPAR) as a biomarker of senescence cells and showed that uPAR-targeting chimeric antigen receptor (CAR) T cells efficiently deplete senescent cells both in vitro and in vivo [288, 289]. Recently, Ming et al. (2025) designed a chimeric peptide 'matchmaker' that binds uPAR to promote immune-mediated clearance of senescent cells [290], further establishing uPAR as a promising molecular target for senescent cell elimination. Similarly, CAR T cells targeting NKG2D, another senescence biomarker, selectively and effectively deplete senescent cells and improve multiple aging-associated pathologies and enhance physical performance in aged mice [291]. GPNMB is also demonstrated to be a biomarker of senescent cells, especially in immune cells [292]. Reduction of GPNMB-positive senescent cells improves multiple aging-associated phenotypes and extends the lifespan of progeroid mice [292]. Senolytic therapies also show promising efficacy for NDDs. For example, depletion of p16^INK4A^-positive senescent astrocytes and microglia using *INK-ATTAC* attenuates gliosis, tau hyperphosphorylation and neurodegeneration, thereby improving cognitive function in *MAPT*^P301S^ PS19 mouse model of tauopathy [239]. Shin et al. (2024) demonstrated that the nanoparticle-based delivery of p16^INK4A^ siRNA attenuates Aβ deposition and microglial senescence surrounding the plaques, thereby improving learning and spatial memory deficits in a mouse model of AD [241]. The senolytic cocktail DQ also has potent senolytic effects on senescent cells. Treatment with DQ significantly depletes autophagy-deficient senescent microglia and improves neuropathology in AD mice [103]. In addition, combination treatment with ABT-263 and DQ maintains blood–brain barrier integrity, reduces SASP production, and alleviates memory deficits by removing peripheral senescent cells [293]. Gonzales et al*.* (2023) have reported the first open-label, phase 1 clinical trial of orally delivered senolytic therapy, DQ, in early-stage symptomatic patients with AD, in which no significant improvement was observed in cognitive function from baseline to post-treatment [294]. In PD mice, removal of senescent microglia using chiral gold nanoparticles carrying anti-B2MG and anti-DCR2 antibodies significantly reduces the CSF level of α-synuclein [295]. Together, removal of senescent immune cells may be a promising anti-aging therapy for NDDs; however, the safety profile of long-term senolytic treatment in humans, particularly regarding immune suppression and cancer risk, remains an open question. Furthermore, longitudinal studies in humans are needed to evaluate the clinical efficacy of senolytic drugs in delaying or preventing the progression of NDDs.

### Improving intercellular communication

Senescent cells express higher levels of PD-L1, an immune checkpoint molecule that binds with PD-1 on immune cells to inhibit immunosurveillance of cytotoxic CD8^+^ T cells or NK cells [136]. Consequently, blockade of PD-1 improves the survival and cytotoxic capacity of CD8^+^ T cells, thereby promoting the clearance of senescent cells expressing PD-L1 [296]. Expanding on this concept, Katsuumi et al. (2024) demonstrated that Canagliflozin, a sodium glucose co-transporter 2 inhibitor, enhances immune-mediated clearance of senescent cells by downregulating PD-L1 expression. This further underscores the pivotal role of PD-L1 blockade in anti-aging therapies [297]. In addition, suppression of PD-L1/PD-1 signaling also improves various aging-related phenotypes in NDDs [136, 298–300]. For example, PD-1 blockade promoted myeloid cell recruitment to brain parenchyma via IFN-γ and alleviated AD pathology and cognitive impairment in 5 × FAD and APP/PS1 mice [298]. Moreover, PD-L1 blockade improved cognitive decline and reduced soluble Aβ_1-42_ levels in *Trem2*^−/−^ 5 × FAD mice [299]. In a mouse model of tauopathy, blockade of PD-L1 increased immunomodulatory monocyte-derived macrophages and reduced cognitive deficits and cerebral pathology [300]. The first-in-human, phase 1 trial of anti-PD-L1 antibody IBC-Ab002 in AD was initiated in 2023 (NCT05551741), with results currently pending. Furthermore, blocking PD-L1 also rescues T cell deficiency caused by small extracellular vesicles containing α-synuclein isolated from in vitro *SNCA*-A53T PD model, highlighting the broader relevance of PD-L1 for treating NDDs [139]. While the blockade of PD-L1/PD-1 signaling offers a promising approach for targeting senescent cells, the long-term effects of widespread immune activation and the risks of autoimmune responses must be carefully evaluated. Additionally, the use of PD-1 inhibitors in the context of aging and NDDs requires further validation in human trials, as much of the current evidence comes from animal models.

In addition to targeting the PD-L1/PD-1 axis, the use of young blood or its components is emerging as a potential strategy for rejuvenating aged tissues. Platelet-derived chemokine platelet factor 4 (PF4) is an anti-aging molecule derived from platelets and its level declines with age [301]. Exogenous PF4 treatment has been shown to restore aged immune system, alleviate neuroinflammation, and improve cognitive function in aged mice [301]. In addition, exposure to young blood reversed gene transcriptional changes in aged HSCs and improved age-associated decline in HSCs [302]. Consistently, small extracellular vesicles from young plasma alleviated senescent phenotypes, improved age-associated functional declines, and extended lifespan of aged mice [303]. Another study also demonstrated the beneficial effects of young blood on aging-associated phenotypes and lifespan [304], yet the underlying mechanisms are not fully understood. Tony Wyss-Coray’s group confirmed the safety, tolerability, and feasibility of infusions of young, fresh frozen plasma in AD patients in 2019 [305], although the therapeutic efficacy was not determined in that study. Future studies should aim to further elucidate the mechanisms underlying the rejuvenating effects of young blood and its components. Investigating the specific signaling pathways and cellular interactions involved could pave the way for more targeted interventions.

### Anti-inflammatory therapy

NLRP3 inflammasomes are a crucial driver of immune aging, contributing to chronic inflammation and immune dysfunction [107, 130, 163, 164, 306]. Studies have shown that inhibition of NLRP3 inflammasome activation promotes thymic lymphopoiesis [163], alleviates premature immunosenescence [164], inhibits innate immune activation and astrogliosis [306], and mitigates diabetic vascular aging and damage [164]. Therefore, targeting the NLRP3 pathway represents a viable therapeutic strategy for rejuvenating the aged immune system [164]. T cells with *TFAM* deficiency accelerate cellular senescence by inducing accumulation of blood cytokines, which resembles inflammaging [127]. Blockade of TNF-α signaling can partially delay premature aging in mice with *TFAM*-deficient T cells [127]. A recent study showed anti-inflammatory effects of TNF-α blockade by Etanercept [307]. In a randomized, placebo-controlled, double-blind phase 2 trial, Etanercept showed a beneficial trend for improving cognitive function of AD patients [308]. Ageing is associated with increased levels of pro-inflammatory my-HSCs and myelopoiesis [309]. Depletion of my-HSCs in aged mice promotes immune rejuvenation, marked by increased naïve T and B cells alongside reduced age-related inflammatory markers [310]. Concomitantly, dysregulation of myeloid PGE₂/EP2R signaling drives maladaptive immune responses, culminating in chronic inflammation and immunosenescence [128, 311]. Notably, suppression of peripheral myeloid PGE_2_/EP2R signaling improves cognition and restores youthful immune functions in aged mice via metabolic reprogramming of myeloid cells [311]. Furthermore, IL-11, a pro-inflammatory cytokine of the IL-6 family, negatively affects aging-associated diseases and lifespan by promoting chronic inflammation [312]. Widjaja et al. (2024) showed that IL-11 deficiency restores age-related immune-metabolic dysfunction and prolongs healthspan and lifespan in mice [312]. Collectively, these findings underscore the therapeutic potential of targeting inflammaging to mitigate age-related decline.

The cGAS-STING pathway plays a central role in triggering immune cell senescence and is a promising therapeutic target for reversing immunosenescence. Inhibition of the cGAS-STING signaling pathway alleviates premature senescence phenotypes of Ataxia-Telangiectasia brain organoids [313], and improves neuropathology and memory deficits in tauopathy mice [314] and other AD mouse models [315, 316]. The cGAS-STING pathway is also activated in MPTP mouse model of PD [272, 317]. Microglial cGAS deletion attenuates neurodegeneration and diminishes pro-inflammatory response in astrocytes and microglia induced by MPTP [317]. Collectively, these findings underscore the potential of suppressing the cGAS-STING signaling pathway as a strategy to attenuate both immunosenescence and NDDs.

### Targeting nutrient-sensing pathways

Activation of the mTOR signaling pathway significantly contributes to immune aging [112–114]. Thus, mTOR inhibitors may effectively reverse immunosenescence during aging [318]. Rapamycin is the most common mTOR inhibitor used in both basic research and clinical settings. Rapamycin and its derivatives significantly improve a multitude of aging-associated phenotypes, such as immune deficiency and cardiovascular dysfunction [318]. In addition to rapamycin, the mTOR inhibitor RAD001 also ameliorates immunosenescence by enhancing immune responses to the influenza vaccine and reducing the percentage of senescent CD4^+^PD-1^+^ and CD8^+^PD-1^+^ T cells [319]. Furthermore, mTOR inhibitors such as RTB101, BEZ235, and RAD001, can upregulate IFN-induced antiviral immune responses and decrease infection rates in older adults [320, 321]. Importantly, mTOR inhibitors have been shown to improve neurodegeneration in mouse models of AD [322], PD [323], and ALS [324], highlighting the potential role of mTOR inhibition for treatment of NDDs. In a phase 2 clinical trial, the treatment with rapamycin was well tolerated and provided reassuring safety in ALS patients, although further clinical trials are needed to examine the clinical efficacy of this drug in ALS patients [325]. Although mTOR inhibitors like rapamycin and RAD001 show considerable promise in reversing immune aging, they may cause side effects such as impaired wound healing, increased susceptibility to infections, and metabolic disturbances. Therefore, it is critical to balance mTOR inhibition to achieve beneficial outcomes without compromising the overall health.

NAD replenishment has shown promise in the treatment of NDDs. It improves motor function of PD patients [326, 327]. In individuals with subjective cognitive decline and mild cognitive impairment (MCI), NAD supplementation is safe and reduces p-tau217 concentrations, although with no improvement in cognitive function [328, 329]. In AD patients, treatment with combined metabolic activators containing NAD reduced AD Assessment Scale-cognitive subscale score and increased hippocampal volumes and cortical thickness compared to the placebo group [330]. However, the lack of cognitive benefit in MCI cohorts highlights the complexity of translating biomarker changes into functional gains and underscores the need for further investigation.

Fasting and caloric restriction (CR) significantly suppress mTOR signaling and activate AMPK, SIRT1 and SIRT3, thereby effectively antagonizing aging-related phenotypes. CR improves thymopoiesis, promotes anti-inflammatory effects on adipose tissues, and extends lifespan [331]. In *C. elegans*, dietary restriction extends lifespan by modulating immune pathways involving p38 MAPK signaling [332]. Specifically, long-term CR partially reverses age-related reductions of NK cells, naïve CD4^+^ and CD8^+^ T cells, as well as granzyme B-secreting T cells in the spleen [333]. Moreover, long-term CR also inhibits the expression of senescent markers in T cells, such as PD-1 and KLRG1, which may be mediated by transcription factors NR4A1 and TOX [333]. Additionally, prolonged fasting enhances stress resistance, self-renewal capacity, and regeneration of HSCs by reducing IGF-1 levels and PKA activity, making CR a viable strategy for rejuvenating the aged immune system [334]. Importantly, the protective effects of CR on NDDs have been demonstrated in preclinical NDD models [335–337], warranting further investigation in clinical setting. Critically, long-term adherence to CR may be difficult for individuals, and the effects of CR on metabolism, bone density, and muscle mass raise concerns on the potential side effects in older adults.

Ketogenic diet is a high-fat, moderate-protein, low-carbohydrate diet, which has been shown to extend the lifespan and healthspan in mice [338]. It induces the production of the ketone body β-hydroxybutyrate, which can inhibit NLRP3 inflammasome activation, thus blocking chronic inflammation and cellular senescence [339]. This diet also significantly expands γδ T cells, enhancing immune responses against influenza virus infection [340]. Consistently, Ryu et al. (2021) revealed that activation of ketogenesis expands protective γδ T cells, suppresses NLRP3 inflammasome activation, and reduces pathogenic monocytes in aged mice [341]. Therefore, ketogenic diet may improve immune aging by inhibiting NLRP3 inflammasome activation. In NDDs, ketogenic diet diminishes neuropathology and ameliorates behavioral deficits [342]. In AD patients, ketogenic diet improved daily function and quality of life in a single-phase, assessor-blinded, two-period crossover trial [343]. In MS patients, ketogenic diet significantly improves body composition, fatigue, depression, sleep quality, and neurological disability in relapsing MS patients [344, 345]. While the ketogenic diet offers substantial benefits in preclinical models, long-term adherence to this diet presents a challenge due to its restrictive nature and potential metabolic side effects. More research is needed to fully understand the broader impact of the ketogenic diet on both immune aging and neurodegeneration in aging individuals.

### Maintaining proteostasis

Spermidine, a natural polyamine that declines with human aging, has shown potential to extend lifespan and mitigate age-related diseases through various mechanisms. Specifically, spermidine significantly reverses senescence of B and T cells by enhancing the expression of TFEB, a transcription factor crucial for regulating autophagy [99, 102]. Interestingly, nutritional spermidine has been shown to enhance memory performance in old adults at risk of dementia [346]. Controversially, in another randomized clinical trial, long-term spermidine supplementation in patients with subjective cognitive decline did not improve memory performance compared with placebo [347].

CMA activity gradually decreases in HSCs with advanced age. Genetic or pharmacological activation of CMA successfully rejuvenates HSCs in old mice and humans [97]. Restoration of CMA activity in T cells also rescues immune deficiency in old mice [100]. Defective induction of proteasome activity is also a characteristic of T cell senescence [101]. These findings underscore the multifaceted roles of macroautophagy, CMA, and proteasome in immune aging and suggest promising avenues for therapeutic intervention to enhance immune function for age-related diseases [348]. Future research should focus on the clinical translation of spermidine supplementation, assessing its safety, efficacy, and optimal dosing in patients with NDDs. Moreover, while autophagy induction appears beneficial for rejuvenating senescent immune cells, excessive or dysregulated autophagy could lead to detrimental effects, such as impaired cellular function or excessive cell death. Future studies should explore whether boosting proteasome activity can specifically rejuvenate senescent immune cells without causing unintended consequences.

### Boosting mitochondrial function

Aberrant accumulation of mitochondria due to mitophagy impairment is a pathological marker of aged HSCs [121]. Administration of urolithin A, a mitophagy modulator, enhances mitochondrial function in aged HSCs, thereby restoring their blood reconstitution capability [121]. Urolithin A supplementation also rejuvenates lymphoid compartments and enhances antiviral immune response in old mice [121]. In AD mouse models, urolithin A supplementation improves learning, memory, and olfactory function by reducing Aβ and tau pathologies [349]. In a manganese-induced parkinsonism mouse model, urolithin A administration mitigated Mn-induced mitochondrial dysfunction and inhibited detrimental microglial activation, along with attenuation of neurobehavioral deficits [350]. Several drugs have shown potential to ameliorate aging-associated mitochondrial dysfunction, including TPP-thiazole [351], controlled-release mitochondrial protonophore [352], elamipretide [353] and L-carnitine [354], yet their potential to rejuvenate the aged immune system remains to be determined. Given the pivotal role of mitochondrial dysfunction in NDDs, reversing this dysfunction has been linked with reduced neurodegeneration and improved behavioral outcomes in preclinical NDD models [355, 356]. Although therapies such as urolithin A have shown promise in preclinical models, the translation of these findings into human therapies presents challenges. Long-term safety and efficacy need to be thoroughly evaluated, especially given the potential complexity of modulating mitochondrial function.

### Reversing epigenetic dysregulation

Recent studies have identified several key anti-immunosenescence targets that could reverse epigenetic dysregulation. These include factors such as cell division control protein 42 (Cdc42) [357], miR-181a [94, 358], SIRT1 [90, 91], Ezh1 [88], Phf6 [89] and SIRT3 [359]. Modulation of these targets can promote the self-renewal capacity of HSCs [88], increase lymphoid differentiation [89], restore epigenetic abnormalities [88, 357], and improve anti-viral innate immunity [94, 358]. Interestingly, the SIRT1 activator resveratrol improves neuronal injury and α-synuclein pathology in PD models [360, 361], and modulates neuroinflammation and improves cognitive function in both AD animal models [362] and AD patients [363]. However, all the above-mentioned anti-immunosenescence targets except SIRT1, have not yet been investigated in the context of NDDs. Consequently, future research should focus specifically on the interplay between immune epigenetics and neurodegeneration in both preclinical and clinical settings.

### Repairing DNA damage

The integrity of nuclear lamina is essential for healthy aging and longevity. The key components of nuclear lamina, such as Lamin A [70, 71, 364], Lamin B1 [4], and Lamin C [364], are promising targets to alleviate aging-associated phenotypes in immune aging. Additionally, the DNA repair machinery system, which includes Per2 [155], Ercc1 [75, 76], MRE11A [73, 365], Nbs1 [77, 78], SIRT6 [366], ATM [79, 366] and DNA-PKcs [367], is also a potential target for reversing immune aging. Given the role of both nuclear lamina integrity and DNA repair in neuronal health, it is plausible that enhancing these pathways could mitigate neurodegenerative processes.

### Telomere maintenance and elongation

The maintenance of telomere length is essential for healthy aging. Lanna et al. (2022) reported that transfer of telomere vesicles to T cells can elongate telomeres independently of telomerase action, thereby providing a promising approach to maintaining telomere integrity [368]. Telomerase reverse transcriptase (TERT) is a component of telomerase and its overexpression has been shown to delay aging and extend lifespan [369]. Interestingly, TERT activation reduces amyloid pathology, restores spine morphology, and improves learning and memory in AD [370]. Additionally, telomerase reactivation also extends telomeres, attenuates DNA damage signaling, and reverses tissue degeneration in aged telomerase-deficient mice [371]. Furthermore, telomerase gene therapy alleviates several aging-associated phenotypes, such as insulin resistance, osteoporosis, and BM aplasia [372]. While telomere-targeted therapies show considerable promise in rejuvenating various tissues, their application in treating NDDs and improving immune aging requires further exploration.

### Rebuilding gut microbiome

FMT has been shown to rejuvenate aged HSCs [194], rescue ageing-associated germinal center deficiency [373], and enhance healthspan and lifespan in progeroid mouse models [191], thereby providing a potential therapy to rejuvenate aged immune system. In addition, microbiota transplantation from young donors into an aged host reverses aging-associated changes in both peripheral and brain immunity and improves cognitive behavior [195]. Furthermore, the transplantation of specific gut bacteria also ameliorates aging-associated phenotypes. *Lactobacillus acidophilus* DDS-1, a probiotic strain, has been shown to increase butyrate levels and suppress the production of inflammatory cytokines such as IL-6, IL-1β, IL-1α, and IFN-γ [374]. Similarly, *Lactobacillus plantarum* GKM3 significantly enhances memory retention, extends longevity, and reduces oxidative stress in SAMP8 mice [375]. Furthermore, supplementation with *Akkermansia muciniphila* markedly alleviates chronic inflammation and immune-related processes in accelerated aging mice [376] and demonstrated anti-inflammatory effects in overweight and obese human volunteers [377]. In elderly humans (over 75 years), supplementation with *Bifidobacterium longum* Bar33 and *Lactobacillus helveticus* Bar13 mixture increased naïve T cells, regulatory T cells, B cells, and NK cell activity and reduced memory T cells compared with placebo, indicating significant anti-immunosenescence effects [378]. Although current studies highlight the beneficial effects of FMT and specific probiotics on both immune and brain aging, the long-term efficacy and safety of FMT, particularly in NDD patients, require further evaluation, as altering the microbiome could have unpredictable effects on neurodegeneration. In PD patients, although FMT demonstrated acceptable safety, its effects on clinical symptoms remain controversial [379, 380]. In a clinical trial in China (ChiCTR1900021405), FMT reduced PD-related autonomic symptoms [379], improved gastrointestinal disorders, and significantly increased the complexity of the microecological system in the patients [379]. However, in a recent larger clinical trial, FMT did not reverse motor decline in PD patients [380]. In sporadic ALS patients, FMT also failed to demonstrate improvement in motor symptoms [381]. The failures of these clinical trials in NDDs may be due to the variability of microbiome composition between individuals, which might influence the outcomes of microbiota-based therapies. Personalized approaches according to individual microbiome profiles may enhance the effectiveness of these therapies.

### Multi-target therapeutics

Metformin, an oral diabetes medication, lowers blood sugar levels by reducing glucose absorption, production, and insulin resistance. It is a widely studied geroprotective drug, exerting pleiotropic effects primarily through activation of AMPK [382]. Metformin-mediated AMPK activation promotes cellular energy homeostasis and metabolic regulation, thereby exerting a critical anti-immunosenescence effect by restoring energy dysmetabolism in senescent immune cells. Importantly, metformin also acts via AMPK-independent pathways, including Nrf2 [383], cGAS-STING signaling [384], and CMA [382]. Therefore, metformin may counteract immunosenescence through complementary biological pathways. Metformin has been shown to inhibit T cell senescence in middle-aged individuals, suggesting a promising strategy for managing age-related diseases [385]. Importantly, metformin has been shown to improve ageing-related phenotypes in multiple immune cells, such as B cells [386], macrophages [387], and monocyte [388]. In a mouse model of PD, metformin significantly delays astrocyte senescence and improves neurodegeneration by reducing mtDNA release through mitofusin 2 and suppressing the cGAS-STING signaling pathway [384]. Metformin improves memory in cognitively normal individuals [389] and lowers the risk of dementia in patients with Type 2 diabetes [390]. Metformin administration significantly reduces Aβ plaque load and chronic inflammation, alleviates spatial memory deficits and neuron loss, and enhances neurogenesis in APP/PS1 mice [382]. Furthermore, metformin also decreases RAN (repeat-associated non-AUG) protein levels, improves behavior and attenuates neuropathology in *C9orf72*-related ALS/FTD mice [391]. The recent study by Yang et al. (2024) provides compelling evidence for the role of metformin in slowing CNS aging, demonstrating an approximate six-year regression in brain aging of non-human primates [383]. In patients with MCI, metformin has been shown to enhance blood perfusion in the orbitofrontal cortex and ameliorate cognitive impairment [392–394], while its effects in AD patients remain underexplored.

Physical exercise, on the other hand, can prevent, limit, or even reverse immunosenescence by restoring the self-renewal capacity of HSCs [395], reducing exhausted/senescent T cells [396], increasing naïve T cells [396], rejuvenating aged microglia [397], lowering circulatory levels of inflammatory cytokines ("inflammaging") [398], and enhancing neutrophil phagocytic activity [399] and vaccination responses [400]. Therefore, physical exercise can improve immune aging by targeting multiple immune cell types. In sedentary adults with obesity, 12-week physical exercise intervention mitigated p16^INK4A^ levels in PBMCs, indicating that physical exercise is a promising strategy to alleviate premature immune senescence [401]. According to recent clinical trials, physical exercise can exert beneficial effects on the motor signs and quality of life of PD patients [402–406]. Physical exercise can also reduce the incidence of AD and exert multi-domain benefits in patients with AD or MCI [407–410]. Consistently, physical exercise improves the behavior and neuropathology in AD and PD mouse models [411, 412]. Physical exercise similarly has beneficial effects on the clinical symptoms of ALS patients [413, 414]. While the positive impact of exercise on immunosenescence and neurodegeneration is well-documented, there is an urgent need to explore the optimal type, intensity, and duration of exercise to yield the greatest benefits for different populations, especially in the elderly and those with NDDs.

Beyond well-established approaches like metformin and physical exercise, other multi-target therapies are emerging as promising strategies for improving immunosenescence and ameliorating NDDs. Among these, traditional Chinese medicine (TCM) has garnered attention for its holistic, multi-component formulations that often target several biological pathways simultaneously [415]. TCM derived from natural products is believed to possess anti-inflammatory, immunomodulatory, and neuroprotective properties [416]. Bacille-Calmette-Guérin (BCG) vaccine may also hold promise for improving immunosenescence and offering potential strategies for the intervention of NDDs. The BCG vaccine, traditionally used as a tuberculosis vaccine, has also been explored for its potential role in improving immune aging. Recent studies suggest that BCG vaccination can induce long-term changes in the immune system, promoting a more robust and responsive immune environment [417]. Clinical evidence has pointed to the potential benefits of BCG vaccination in lowering the risk of AD and reducing the progression of certain neurodegenerative conditions through its effects on immune regulation [418, 419]. In conclusion, multi-target approaches like TCM and BCG vaccination hold promise as adjunctive therapies to existing interventions for immunosenescence and NDDs, highlighting the need for highly controlled, multi-center trials.

## Conclusion

Collectively, immunosenescence is a key component of the normal aging process, affecting various immune cell populations, including HSCs, immune progenitors, and fully differentiated immune cells distributed across different tissues and organs. In this review, we propose 11 molecular hallmarks of immunosenescence, offering new insights into the underlying biology of immune aging. Immunosenescence is evident in both normal brain aging and NDDs, and has a strong impact on the natural course of brain aging and pathogenesis of NDDs, thereby serving as a novel therapeutic target to delay brain aging and ameliorate neurodegeneration. We propose 12 hallmark-targeting therapies aimed at rejuvenating the aged immune system. These therapies could serve as a valuable guide for the future development of preventive and personalized medicine for elderly individuals and patients with NDDs. By addressing key hallmarks of immunosenescence, such as reducing chronic inflammation, restoring mitochondrial function, and promoting immune cell renewal, these interventions have the potential to extend healthspan, prolong longevity, and improve quality of life in aging populations.

## Data Availability

Not applicable.

## Abbreviations

ABCs: Age-associated B cells
AD: Alzheimer’s disease
ALS: Amyotrophic lateral sclerosis
AP-1: Activator protein-1
AMD: Age-related macular degeneration
AMPK: AMP-activated protein kinase
ATM: Ataxia telangiectasia mutated
Aβ: β-Amyloid
BAMs: Border-associated macrophages
BM: Bone marrow
CAR: Chimeric antigen receptor
cGAS: Cyclic GMP-AMP synthase
CMA: Chaperone-mediated autophagy
CR: Caloric restriction
DAM: Disease-associated microglia
DSBs: Double-strand breaks
EAE: Experimental autoimmune encephalomyelitis
FMT: Fecal microbiota transplantation
FTD: Frontotemporal dementia
HSCs: Hematopoietic stem cells
IFN-γ: Interferon-γ
IGF1: Insulin-like growth factor-1
KLRG1: Killer cell lectin-like receptor G1
LN: Lymph node
MAPK: Mitogen-activated protein kinase
MS: Multiple sclerosis
mTOR: Mechanistic target of rapamycin
NAD^+^: Nicotinamide adenine dinucleotide
NLRP3: NOD-, LRR- and pyrin domain-containing protein 3
NK: Natural killer
NDD: Neurodegenerative disease
PD: Parkinson’s disease
Phf6: Plant homeodomain 6
PI3K: Phosphoinositide 3-kinase
MCM: Mini-chromosome maintenance
RICTOR: Rapamycin-insensitive companion of mTOR
SA-β-Gal: Senescence-associated β-galactosidase
SASP: Senescence-associated secretory phenotype
STING: Stimulator of interferon genes
S6K: S6 kinase
TCR: T cell receptor
TCM: Traditional Chinese medicine
TEM: T effector memory
TFEB: Transcription factor EB
TERT: Telomerase reverse transcriptase
uPAR: Urokinase-type plasminogen activator receptor
UPS: Ubiquitin–proteasome system
